# Trem2 activation by renal tubular debris sustains Arg1^+^ macrophage survival and promotes tubular epithelial repair in renal ischemia–reperfusion injury

**DOI:** 10.3389/fimmu.2026.1819941

**Published:** 2026-04-10

**Authors:** Jiayu Wang, Zhiyi Zhao, Runyi Wang, Xin Zheng, Xiaopeng Hu

**Affiliations:** 1 Department of Urology, Beijing Chao-Yang Hospital, Capital Medical University, Beijing, China; 2 Institute of Urology, Capital Medical University, Beijing, China

**Keywords:** IRI, macrophages, tubular cells, Arg1, Trem2, efferocytosis, survival

## Abstract

Ischemia-reperfusion injury (IRI) severely impairs the function of native and transplanted kidneys, with Arg1^+^ macrophages playing a pivotal role in tubular regeneration. The triggering receptor expressed on myeloid cells 2 (Trem2) mediates debris clearance by recognizing lipids from injured cells, but its role in Arg1^+^ macrophage survival remains unclear. Here, we show that Trem2 not only regulates the phagocytic activity of Arg1^+^ macrophages but also sustains their survival. Single-cell transcriptomics revealed marked Trem2 upregulation in macrophages after IRI, predominantly in the *Arg1^hi^Ecm1^hi^* ECM-Remodeling Mac subset. Trajectory analysis indicated coordinated Trem2 and Arg1 expression during macrophage differentiation, while intercellular signaling analysis showed that IRI enhanced Apoe-Trem2–mediated communication among *Arg1^hi^Ecm1^hi^* ECM-Remodeling Mac, promoting debris clearance. *In vivo*, Trem2^+^Arg1^+^F4/80^+^ macrophages abundantly infiltrated the kidney early after IRI, whereas inhibition of Trem2 aggravated kidney injury and further impaired kidney function. *In vitro*, tubular debris induced Trem2 expression and proliferation in Arg1^+^ macrophages, whereas Trem2 knockdown or pharmacological inhibition of Trem2 reduced proliferation, increased apoptosis, and impaired their ability to support tubular epithelial repair via paracrine polyamines, EGF, and VEGF. Transcriptomic analysis further showed that Trem2 knockdown upregulated Pten and suppressed the anti-apoptotic protein Bcl2 in Arg1^+^ macrophages, thereby reducing cell viability, while a Pten inhibitor VO-Ohpic reversed these effects. Together, these findings identify a previously unrecognized mechanism by which tubular debris sustains Arg1^+^ macrophage survival via Trem2 activation and support targeting Trem2 and downstream PI3K-AKT signaling to enhance macrophage-mediated kidney repair.

## 1 Introduction

Ischemia–reperfusion injury (IRI) is a major cause of acute kidney injury (AKI) and a critical determinant of kidney transplant survival (1). The incidence of AKI is estimated at 10–15% among hospitalized patients and rises to approximately 30% in critically ill individuals, with associated mortality reaching 10–20% (2, 3). IRI represents the predominant etiology of AKI, typically arising from renal hypoperfusion induced by shock or surgical clamping of the renal vasculature (4, 5). Although AKI is often self-limiting, inadequate intervention can drive progression to chronic kidney disease (CKD) (6). In transplantation, IRI is an unavoidable pathological process that frequently results in delayed graft function (DGF) (7). However, effective therapeutic strategies against IRI remain lacking, underscoring the urgent need to identify novel targets for intervention.

Following tissue injury, macrophages upregulate arginase-1 (Arg1), which catalyzes the conversion of arginine to ornithine and urea. Ornithine is further metabolized into polyamines that promote cell growth and proliferation, and these metabolites can be released in a paracrine manner to facilitate tubular epithelial cell regeneration and repair after IRI (8). Arg1^+^ macrophages also secrete extracellular matrix (ECM) components such as Fn1 and Spp1, thereby contributing to ECM remodeling (9, 10). Triggering receptor expressed on myeloid cells 2 (Trem2) is a single-pass transmembrane immune receptor, highly expressed in macrophages, that regulates their proliferation, apoptosis, and phagocytic activity (11–13). Previous studies have shown that Trem2 recognizes damage-associated lipids and exerts dual functions in neurodegenerative disease: maintaining microglial clearance of amyloid-β and injury sensing, while simultaneously acting as a co-stimulatory molecule to support microglial survival (14). Trem2 alleviates acute lung injury in mice and improves valve calcification by reducing the release of inflammatory cytokines from macrophages, inhibiting ferroptosis, and mitigating pyroptosis (15, 16). Moreover, the deficiency of Trem2 in macrophages suppresses their M2 polarization (17). More recently, Trem2 on macrophages has been reported to activate the PI3K-AKT pathway and exert strong renoprotective effects during IRI. Functionally, Trem2-overexpressing macrophages attenuate post-IRI inflammation and the AKI-to-CKD transition, mirroring the exacerbated injury, fibrosis, and inflammation in Trem2-deficient mice (18, 19). During IRI, tubular epithelial cells undergo necrosis and apoptosis as a result of mitochondrial oxidative stress, generating abundant debris enriched in phospholipids and oxidized phospholipids (20, 21), These debris may serve as Trem2 ligands, thereby enhancing macrophage-mediated phagocytosis and clearance. Yet, whether Trem2 also sustains the survival of Arg1^+^ macrophages during IRI to preserve their regenerative capacity remains unclear. Our study focused on how Trem2 influences the survival of Arg1^+^ macrophages upon tubular debris stimulation, uncovering a previously unrecognized protective mechanism of Trem2 in IRI.

Here, by integrating single-cell transcriptomic data from mouse kidneys at multiple time points after IRI, we reveal that *Trem2* is robustly upregulated in *Arg1^hi^Ecm1^hi^* ECM-Remodeling Mac. We further show that Trem2 mediates Apoe-Trem2 interactions that promote the phagocytosis of tubular epithelial debris. *In vivo*, Trem2 and Arg1 are co-expressed in macrophages infiltrating the kidney after IRI, whereas pharmacological inhibition of Trem2 with TREM2-IN-1 aggravated early kidney injury and further impaired kidney function in IRI mice. *In vitro*, tubular debris markedly induces Trem2 expression in Arg1^+^ macrophages and enhances their proliferative activity, and these surviving Arg1^+^ macrophages thereby facilitate tubular repair through the paracrine release of polyamines, HGF, and VEGF. Whereas Trem2 knockdown or pharmacological inhibition of Trem2 suppresses proliferation, increases apoptosis, and impairs their capacity to promote tubular epithelial repair. Mechanistically, Trem2 knockdown or pharmacological inhibition of Trem2 activates Pten and suppresses the expression of Bcl2, a downstream anti-apoptotic protein of the PI3K–AKT pathway, ultimately driving Arg1^+^ macrophage apoptosis. Treatment with the Pten inhibitor VO-Ohpic restored Bcl2 expression and increased the survival of Arg1^+^ macrophages. Collectively, these findings identify a Trem2-mediated “lipid sensing–survival signaling” mechanism as essential for maintaining the reparative function of Arg1^+^ macrophages during IRI. Targeting Trem2 or modulating its downstream PI3K-AKT signaling may therefore represent a promising therapeutic approach to preserve macrophage survival in the early phase of IRI, promote tubular regeneration, and improve kidney outcomes.

## 2 Methods

### 2.1 Mice

All animal procedures in this study were examined and approved by the Animal Welfare and Ethics Committee of Beijing Chao-Yang Hospital, Capital Medical University (Approval No. 24-2014). C57BL/6N male mice (8–10 weeks old, 23-26g) were obtained from the specific pathogen-free (SPF) facility at Capital Medical University. The mice were housed in a pathogen-free environment under a 12-hour light/dark cycle. General anesthesia was induced via intraperitoneal (i.p.) injection ketamine (80–100 mg/kg; Cayman Chemical) and xylazine (10 mg/kg; Selleck Chemicals); euthanasia was performed by cervical dislocation under deep anesthesia. Efforts were made to minimize animal suffering and reduce the number of animals used, adhering to the ARRIVE guidelines for transparent and rigorous animal research reporting (22).

### 2.2 Renal ischemia–reperfusion injury model

Male C57BL/6N mice were used for IRI model establishment. Following anesthesia, right nephrectomy was performed via a 10-mm dorsal incision after pedicle ligation (3–0 suture). The left renal pedicle was clamped for 30 min to induce ischemia, confirmed by color change. Body temperature was maintained at 36–37°C using a heating pad and saline-moistened gauze with continuous monitoring. Upon clamp removal, reperfusion was visually verified, and the incision sutured in layers. In addition to the IRI and sham groups, a TREM2 inhibitor–treated group was included. Mice in this group received the TREM2 inhibitor TREM2-IN-1 (HY-160421, MedChemExpress) (23), which was administered via intraperitoneal injection prior to surgery at a dose of 1.5 mg/kg. Postoperative care included ibuprofen (200 µg/ml in drinking water) administered before and up to 48 hours after surgery. Mice had free access to food and water in a temperature-controlled environment. Sham-operated controls underwent the same procedures without vascular clamping. At 1 d, 3 d, 7 d, and 14 d post-injury, kidney and serum samples were collected from all experimental groups, including the TREM2 inhibitor–treated group.

### 2.3 Preparation of tubular epithelial cell debris and RAW264.7 cell culture

TCMK-1 cells (ATCC, CCL-139) were cultured in high-glucose DMEM (Servicebio, G4511) supplemented with 10% FBS and 1% penicillin/streptomycin. After reaching adherence, the cells were scraped from the culture dishes using a pre-chilled cell scraper, resuspended in ice-cold PBS, and subjected to three cycles of rapid freezing in liquid nitrogen (3–5 minutes) and thawing in a 37°C water bath until completely melted. After the final cycle, renal tubular cell debris were collected for subsequent experiments. RAW264.7 (The mouse macrophage cell line) cells were purchased from Procell Life Science&Technology Co., Ltd (CL-0190) and cultured in RAW264.7 cell-specific medium (Procell, CM-0190). RAW264.7 cells were treated with 20 ng/ml IL-4 protein (MedChemExpress, HY-P70653) for 24 hours to induce polarization. TCMK-1 cell debris were quantified under a microscope and added to 24-well plates at a density of 1 × 10^4^ to 2 × 10^4^ debris per well. RAW264.7 cells (8 × 10^4^ cells per well in 24-well plates) were then co-cultured with the debris for 24 hours under standard conditions (37°C, 5% CO_2_).

### 2.4 Construction of stable Trem2 knockdown RAW264.7 cells

A lentiviral vector carrying shRNA targeting Trem2 knockdown and GFP was used to transduce RAW264.7 cells. The sequences of the shRNAs and the negative control (NC) were as follows: mTrem2-shRNA1: CCGG-ctgcgttctcctgagcaagtt-CTCGAG-aacttgctcaggagaacgcag-TTTTTT; mTrem2-shRNA2: CCGG-gagcacagtcatcgcagatga-CTCGAG-tcatctgcgatgactgtgctc-TTTTTT; and shNC: CCGG-GATTCTCCGAACGTGTCACGT-CTCGAG-ACGTGACACGTTCGGAGAATC-TTTTTT. To enhance infection efficiency, 5 μg/mL of polybrene was added during the infection process. After 48 hours of infection, the medium was replaced with fresh culture medium and cells were further incubated. Subsequently, 2 μg/mL of puromycin was added for selection, and the selection was carried out for 7 days until only successfully transduced cells remained. During the selection process, GFP signals were monitored using fluorescence microscope to confirm transduction efficiency. The knockdown level of Trem2 in the RAW264.7 cells was validated by qPCR and Western blot, resulting in the establishment of a stable transduced cell line.

### 2.5 BMDM culture and TREM2-IN-1 intervention

Primary mouse bone marrow–derived macrophages (BMDMs) were purchased from Fuheng Biology (FH-M078, Shanghai, China). Upon arrival, cells were cultured strictly according to the manufacturer’s instructions and used for downstream experiments under physiological conditions or after stimulation with renal tubular cell debris. BMDMs were seeded using the same plating protocol as described in Section 2.3. In the renal tubular cell debris–stimulated group, the TREM2 inhibitor TREM2-IN-1 (HY-160421, MedChemExpress) was added at a concentration of 5 μM to assess cell proliferative activity and apoptosis.

### 2.6 Conditioned medium experiments

IL-4–induced Arg1^+^ macrophages (8 × 10^4^ cells per well in 24-well plates), including shNC and Trem2 knockdown (shTrem2 #1 and shTrem2 #2) macrophages, as well as Trem2 inhibitor TREM2-IN-1–treated Arg1^high^ BMDMs, were cultured under standard conditions and stimulated with 2 × 10^4^ renal tubule debris per well for 24 h. The culture supernatant was then collected, centrifuged at 1,200 × g for 10 min to remove cellular debris. The resulting conditioned medium was applied to TCMK-1 cells (1 × 10^5^ cells per 10 cm dish) for 24 h to assess cell number and proliferative activity using the CCK-8 assay. Conditioned medium from untreated culture medium was prepared in parallel as a control.

### 2.7 RNA extraction, Library preparation and sequencing

Total RNA was extracted using TRIzol^®^ Reagent following the manufacturer’s instructions. RNA purity (OD260/280 = 1.8–2.2) was assessed with a NanoDrop spectrophotometer, and integrity was evaluated using a Bioanalyzer. Only high-quality RNA samples were used for library construction. RNA purification, reverse transcription, library preparation, and sequencing were conducted at Shanghai Majorbio Bio-pharm Biotechnology Co., Ltd. (Shanghai, China) following Illumina’s protocol (San Diego, CA). RNA-seq libraries were generated using the Illumina^®^ Stranded mRNA Prep Ligation kit with 1 μg of total RNA. mRNA was enriched using oligo(dT) beads via polyA selection, fragmented, and reverse-transcribed into double-stranded cDNA using the SuperScript kit (Invitrogen) and random hexamer primers. The resulting cDNA was end-repaired, phosphorylated, and A-tailed as per standard procedures. Target fragments (~300 bp) were size-selected on 2% agarose gel, then amplified with Phusion DNA polymerase (NEB) for 15 cycles. Libraries were quantified using Qubit 4.0 and sequenced as paired-end reads (2 × 150 bp) on the Illumina NovaSeq X Plus. Raw paired-end reads were processed for adapter trimming and quality filtering using fastp (24) with default settings. Clean reads were then aligned to the reference genome in orientation-aware mode using HISAT2 (25). Subsequently, mapped reads from each sample were assembled into transcripts with StringTie, based on the reference annotation. The resulting transcript assemblies were then used for downstream analyses, including gene expression quantification, differential expression analysis, functional enrichment, protein-protein interaction (PPI) analysis and transcription factor (TF) binding site prediction.

### 2.8 Publicly transcriptome datasets acquisition

To investigate the single-cell level changes in the mouse kidney following IRI, we selected datasets with a 30-minute ischemia duration as our source of publicly available single cell and bulk RNA-seq data after systematic search in the GEO and OMIX databases. Samples at different time points following IRI were collected. The details of these datasets were summarized in **Table 1**.

**Table 1:** Datasets used in the current study.

Accession number	Species	Data type	Platform	Time post IRI	Publication
GSE171639	Mus musculus	scRNA-seq	Illumina NovaSeq 6000	Sham	(26)
				6 hours	
OMIX004421	Mus musculus	scRNA-seq	Illumina HiSeq X Ten	Sham	(27)
				1day	
				3 days	
GSE139506	Mus musculus	scRNA-seq	Illumina HiSeq 2500	Sham	(28)
				1 day	
				2 days	
				4 days	
				7 days	
				11 days	
				14 days	
GSE180420	Mus musculus	scRNA-seq	Illumina HiSeq 4000	Sham	(29)
				1 day	
				3 days	
				14 days	
GSE131685	Homo Sapiens	scRNA-seq	Illumina HiSeq X Ten	Control (3) (Chinese)	(30)
GSE174220	Homo Sapiens	scRNA-seq	Illumina HiSeq X Ten	AKI (2) (Chinese)	-–
KPMP	Homo Sapiens	scRNA-seq	10x Genomics Chromium v3	Control (28) and AKI (14) (Western)	(31)

### 2.9 Additional materials and methods

Detailed methods and analyses for the remaining experiments are available in the online **Supplementary Materials**.

## 3 Results

### 3.1 Single-cell transcriptomic analysis reveals the temporal dynamics of *Arg1^hi^Ecm1^hi^* ECM-remodeling macrophages subpopulations during renal repair following IRI

**Figure 1:**
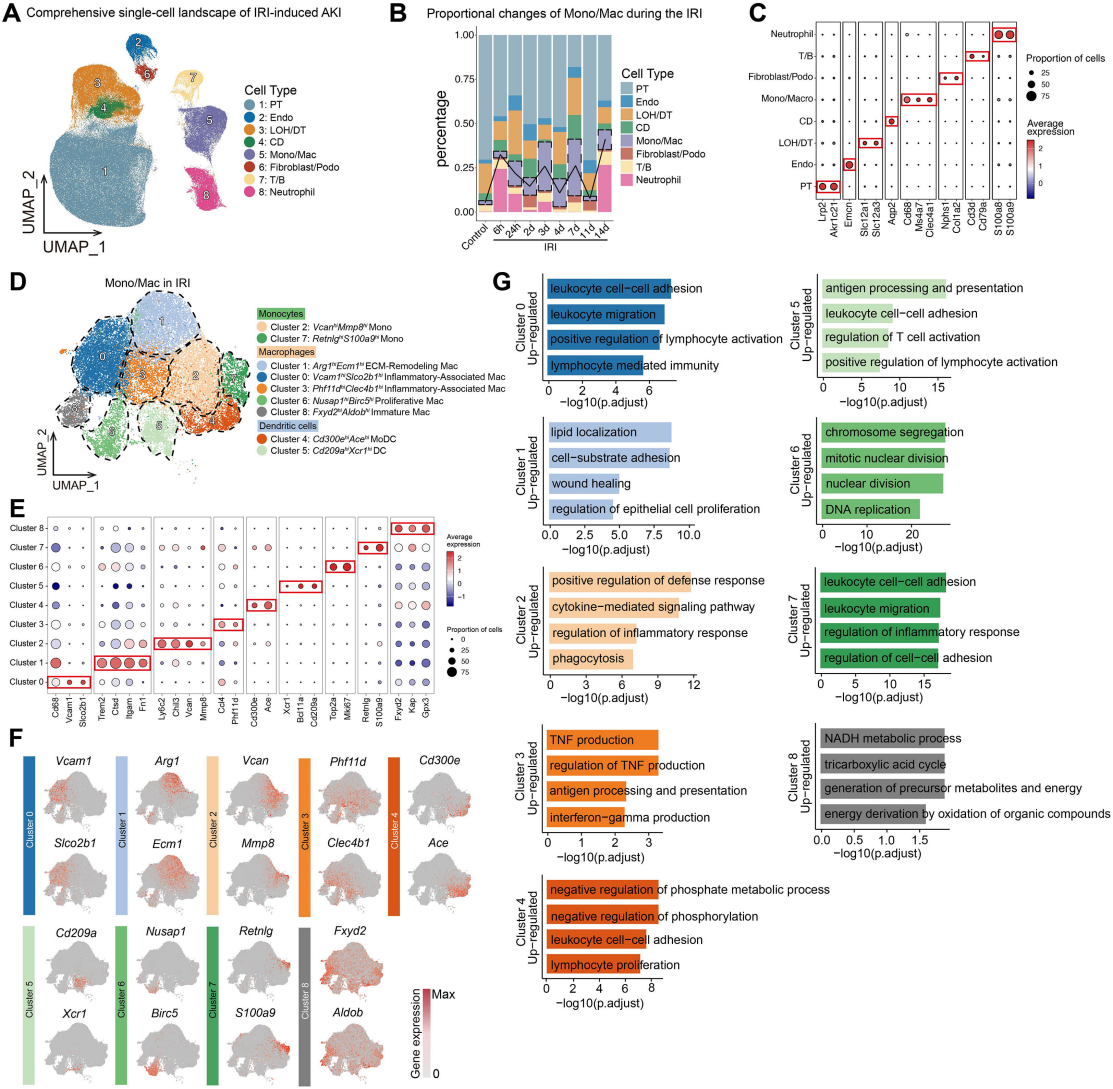
Single-cell transcriptomic analysis of renal IRI identifies temporal dynamics of *Arg1^hi^Ecm1^hi^* ECM-Remodeling Macrophages. **(A)** UMAP embedding of 243,350 renal single cells identifying eight major populations, including proximal tubule (PT), endothelial (Endo), loop of Henle/distal tubule (LOH/DT), collecting duct (CD), monocyte/macrophage (Mono/Mac), fibroblast/podocyte (Fibroblast/Podo), T/B cells and neutrophils. **(B)** Temporal changes in cell type composition showing expansion of Mono/Mac populations after IRI. **(C)** Bubble plot of representative marker genes defining major cell types. **(D)** UMAP embedding of 25,343 monocytes/macrophages resolved into 10 clusters comprising three monocyte, six macrophage and one dendritic cell (DC) subsets. **(E, F)** Expression of representative marker genes delineating *Vcam1^hi^Slco2b1^hi^* inflammatory-associated Mac, *Arg1^hi^Ecm1^hi^* ECM-Remodeling Mac, *Nusap1^hi^Birc5^hi^* proliferative Mac, *Fxyd2^hi^Aldob^hi^* immature Mac and other subsets. **(G)** GO enrichment analysis of DEGs in nine macrophage subpopulations.

We comprehensively integrated the renal single-cell transcriptomic data from an IRI-induced AKI model, resulting in the identification of 243,350 high-quality cells following quality control. UMAP dimensionality reduction and clustering analysis revealed 8 major cell populations, including proximal tubule cells (PT), endothelial cells (Endo), loop of Henle/distal tubule cells (LOH/DT), collecting duct cells (CD), monocyte/macrophages (Mono/Mac), fibroblast/podocytes (Fibroblast/Podo), T/B cells (T/B), and neutrophils (**Figure 1A**). Dynamic analysis of cell proportions showed a marked temporal dependence for the Mono/Mac cell population during the IRI process. In the control group, Mono/Mac cells constituted only 2.0% of the total cell population, but by 24 hours post-IRI, their proportion rapidly increased to 13.9%, and reached 27.8% and 31.9% at 3 and 7 days, respectively (**Figure 1B**, **Supplementary Figure 1A**). These results suggest a temporal regulatory mechanism for monocyte/macrophage subpopulations during IRI. The bubble plot illustrates the marker genes for different cell subtypes, including PT (*Lrp2^hi^*, *Akr1c21^hi^*), Endo (*Emcn^hi^*), LOH/DT (*Slc12a1^hi^*, *Slc12a3^hi^*), CD (*Aqp2^hi^*), Mono/Mac (*Cd68^hi^*, *Ms4a7^hi^*, *Clec4a1^hi^*), Fibroblast/Podo (*Nphs1^hi^*, *Col1a2^hi^*), T/B (*Cd3d^hi^*, *Cd79a^hi^*), Neutrophil (*S100a8^hi^*, *S100a9^hi^*) (**Figure 1C**).

To further investigate the differentiation process and heterogeneity of monocyte/macrophage subpopulations at different time points post-IRI, we analyzed 25,343 monocytes/macrophages and identified 10 distinct clusters through dimensionality reduction and clustering analysis. These included 3 monocyte clusters (Cluster 2, Cluster 7, Cluster 9), 6 macrophage clusters (Cluster 0, Cluster 1, Cluster 3, Cluster 4, Cluster 6, Cluster 8), and dendritic cells (Cluster 5) (**Supplementary Figure 1B**, **Figure 1D**). Cluster 2, Cluster 7, and Cluster 9 all highly expressed monocyte-specific marker genes such as *Fsfgr3*, *Ly6C2*, and *Chil3*, and were therefore named *Vcan^hi^Mmp8^hi^* Mono (Cluster 2), *Retnlg^hi^S100a9^hi^* Mono (Cluster 7), and *Prf1^hi^Gzma^hi^* Mono (Cluster 9) (32–34). Among the 6 macrophage clusters, Cluster 0 was characterized by high expression of *Vcam1*, *Slco2b1*, and inflammatory markers including *Ccl4*, *Ccl3*, and *Ccl12*, and was designated *Vcam1^hi^Slco2b1^hi^* Inflammatory-Associated Mac (10, 27, 35). Cluster 1 exhibited high expression of tissue repair markers such as *Arg1 (*36–38) and extracellular matrix (ECM) components like *Fn1* and *Ecm1 (*39, 40), alongside the expression of *Trem2*, which contributes to the phagocytosis of cellular debris and promotes tissue repair, thus it was defined as *Arg1^hi^Ecm1^hi^* ECM-Remodeling Mac (10, 41, 42). Cluster 3 showed high expression of *Prf11d*, *Clec4b1*, as well as inflammatory markers such as *Ccl4* and *Ccl3*, and was named *Phf11d^hi^Clec4b1^hi^* Inflammatory-Associated Mac (43, 44). Cluster 4 exhibited high expression of *Cd300e* and *Ace*, markers for monocyte-derived dendritic cells (MoDC) (3, 4). Cluster 6 was enriched for cell cycle-associated genes (*Nusap1*, *Birc5*, *Mki67*, *Top2a*), and was designated *Nusap1^hi^Birc5^hi^* Proliferative Mac (10). Cluster 8 expressed high levels of *Fxyd2*, *Aldob*, and macrophage maturation markers such as *Csf1r*, *Cebpb*, and *Itga4*, and was categorized as *Fxyd2^hi^Aldob^hi^* Immature Mac (10, 45, 46). Cluster 5 represented dendritic cells with high expression of *Cd209a* and *Xcr1* (**Figures 1D–F**).

Cell proportion analysis revealed that the *Arg1^hi^Ecm1^hi^* ECM-Remodeling Mac was most abundant on day 3 post-IRI (**Supplementary Figure 1C**), Given known dissociation and multi-dataset integration biases in single-cell workflows, these observations should be interpreted as qualitative trends rather than precise tissue-level frequencies. GO enrichment was performed on differentially expressed genes (DEGs) from all nine macrophage subpopulations. In particular, the *Arg1^hi^Ecm1^hi^* ECM-remodeling Mac (cluster 1) showed enrichment for processes related to lipid localization, cell–substrate adhesion, wound healing, and regulation of epithelial cell proliferation. Although these cells resemble previously reported M2-like or ECM-remodeling macrophages, they exhibited a pronounced emphasis on lipid metabolic pathways, suggesting an enhanced involvement of lipid handling and signaling during tissue repair (**Figure 1G**). These findings suggest that *Arg1^hi^Ecm1^hi^* ECM-Remodeling Mac plays a critical role in renal tissue repair following IRI, with its activation closely correlated to the timing of IRI. Their activation peaks particularly on day 3 post-IRI, suggesting a broad involvement in the repair of injured renal tubular epithelial cells. Additionally, the repair function of these cells may be related to lipid metabolic signaling. Although our single-cell analysis has characterized the complex transcriptomic heterogeneity of monocytes and macrophages in the IRI model, further investigation is required to determine whether these computationally defined subpopulations possess distinct biological functions.

### 3.2 Trem2, Msr1, and Spp1 are co-upregulated in *Arg1^hi^Ecm1^hi^* ECM-remodeling macrophages at multiple time points following IRI

**Figure 2:**
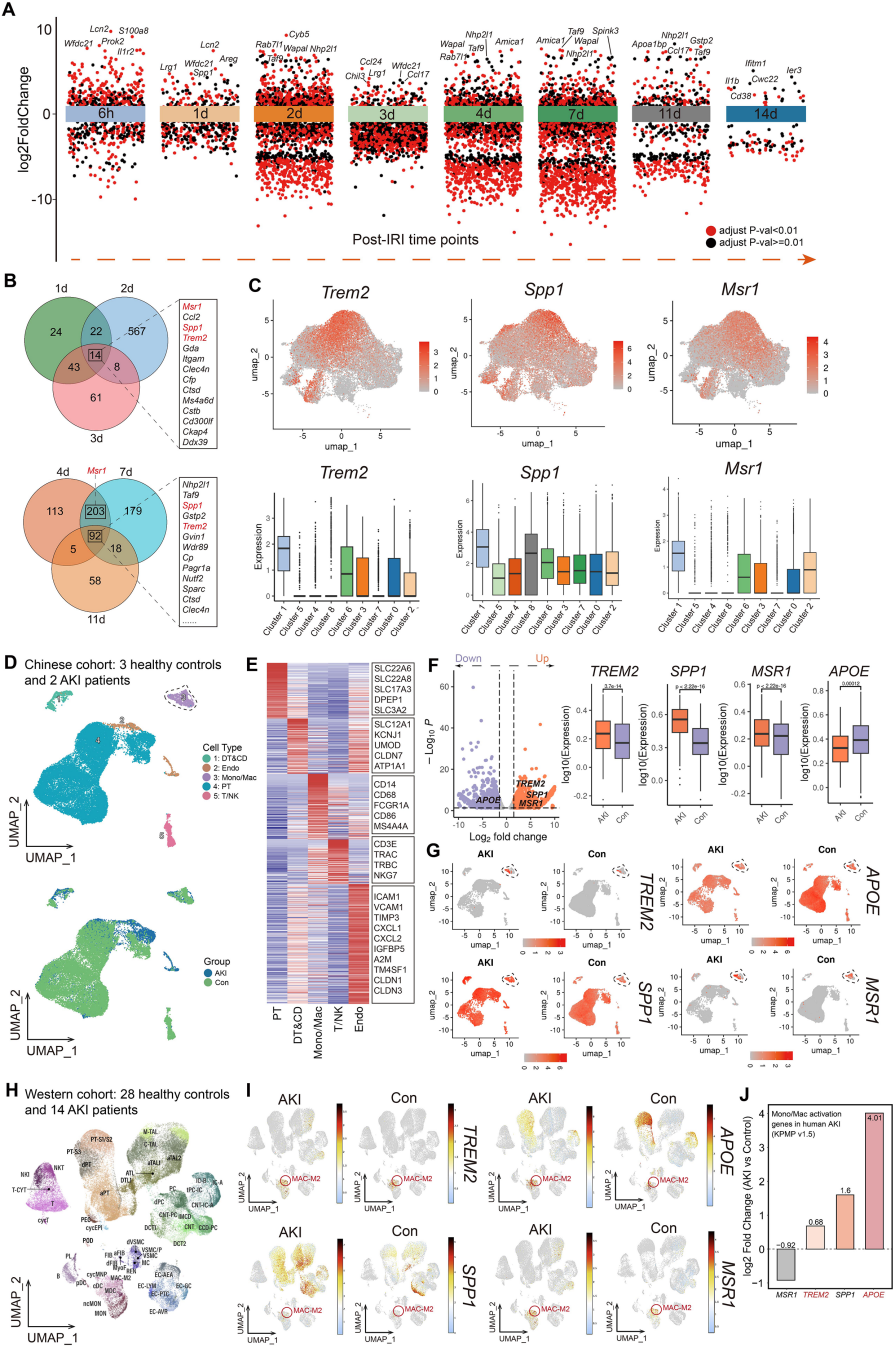
Trem2, Msr1, and Spp1 are co-upregulated in *Arg1hiEcm1hi* ECM-Remodeling Macrophages following IRI and in human AKI kidneys.**(A)** Differential expression analysis of monocyte/macrophage subsets across multiple time points after IRI reveals marked temporal heterogeneity in gene expression profiles. **(B)** *Trem2 and Spp1* display sustained upregulation during both the early (days 1–3) and intermediate-to-late (days 4–11) phases post-IRI, whereas *Msr1* expression is not significantly elevated at day 11. **(C)** FeaturePlot analysis shows that *Trem2, Msr1*, and *Spp1* are predominantly expressed in *Arg1hiEcm1hi* ECM-Remodeling Mac (Cluster 1). **(D)** Dimensionality reduction and clustering of a publicly available human kidney single-cell RNA-seq dataset identify five major populations: distal tubule & collecting duct (DT&CD), endothelial cells (Endo), monocytes/macrophages (Mono/Mac), proximal tubules (PT), and T/NK cells. **(E)** Canonical marker gene expression delineates each cluster. **(F)** Within the monocyte/macrophage population, *TREM2, MSR1*, and *SPP1* are markedly upregulated in AKI kidneys, whereas *APOE* remains unchanged. **(G)** *TREM2* and *MSR1* are specifically restricted to the monocyte/macrophage compartment, while SPP1 and APOE are also induced in renal tubular epithelial and other immune cells following AKI. **(H)** KPMP database analysis of Western individuals (28 healthy controls, 14 AKI). **(I, J)** Transcriptional levels of *TREM2, APOE, SPP1*, and *MSR1* in monocyte/macrophage subsets. *TREM2* is enriched in M2 macrophages and upregulated in AKI alongside *APOE* and *SPP1*, with *MSR1* unchanged.

Differential expression analysis of monocyte/macrophage subpopulations across multiple time points after IRI revealed substantial temporal heterogeneity in gene expression profiles (**Figure 2A**). Interestingly, both *Trem2* and *Spp1* exhibited sustained upregulation during the early (days 1–3) and intermediate-to-late (days 4–11) phases post-IRI, whereas *Msr1* was not significantly upregulated at day 11 (**Figure 2B**). FeaturePlot analysis showed that *Trem2*, *Msr1*, and *Spp1* were predominantly enriched in *Arg1^hi^Ecm1^hi^* ECM-Remodeling Macrophages (Cluster 1) (**Figure 2C**), suggesting that these genes may function cooperatively within this subset to facilitate the clearance of necrotic tubular debris and promote epithelial regeneration following IRI. To investigate the clinical relevance of these findings, we further analyzed a publicly available single-cell RNA-seq dataset from Chinese human kidneys with AKI. Dimensionality reduction and clustering identified five major cell populations: DT&CD, endothelial cells (Endo), monocytes/macrophages (Mono/Mac), proximal tubules (PT), and T/NK cells (**Figure 2D**). The expression profiles of canonical marker genes for each cluster are shown in **Figure 2E**. Differential expression analysis within the monocyte/macrophage population revealed marked upregulation of *TREM2*, *MSR1*, and *SPP1* in AKI kidneys, whereas *APOE* (a major ligand of TREM2) expression remained unchanged (**Figure 2F**). Notably, TREM2 and MSR1 were specifically expressed in the monocyte/macrophage compartment, while *SPP1* and *APOE* were also induced in renal tubular epithelial and other immune cells following AKI (**Figure 2G**). Collectively, these data indicate that *TREM2* and *MSR1* are specifically upregulated in human renal monocyte/macrophage subsets during early AKI, potentially contributing to the phagocytic clearance of tubular debris and modulation of epithelial cell proliferation, akin to the mechanism observed in murine models. We further analyzed 28 healthy control samples and 14 AKI samples from Western individuals in the KPMP database (**Figure 2H**). *Trem2* was predominantly expressed in the M2 macrophage subset. In AKI samples, the transcriptional levels of *Trem2*, *Apoe*, and *Spp1* were markedly elevated in the monocyte/macrophage populations, whereas *Msr1* expression remained unchanged (**Figures 2I, J**).

### 3.3 *Trem2* and *Apoe* cooperate in regulating lipid metabolism pathways in *Arg1^hi^Ecm1^hi^* ECM-remodeling macrophages after IRI

**Figure 3:**
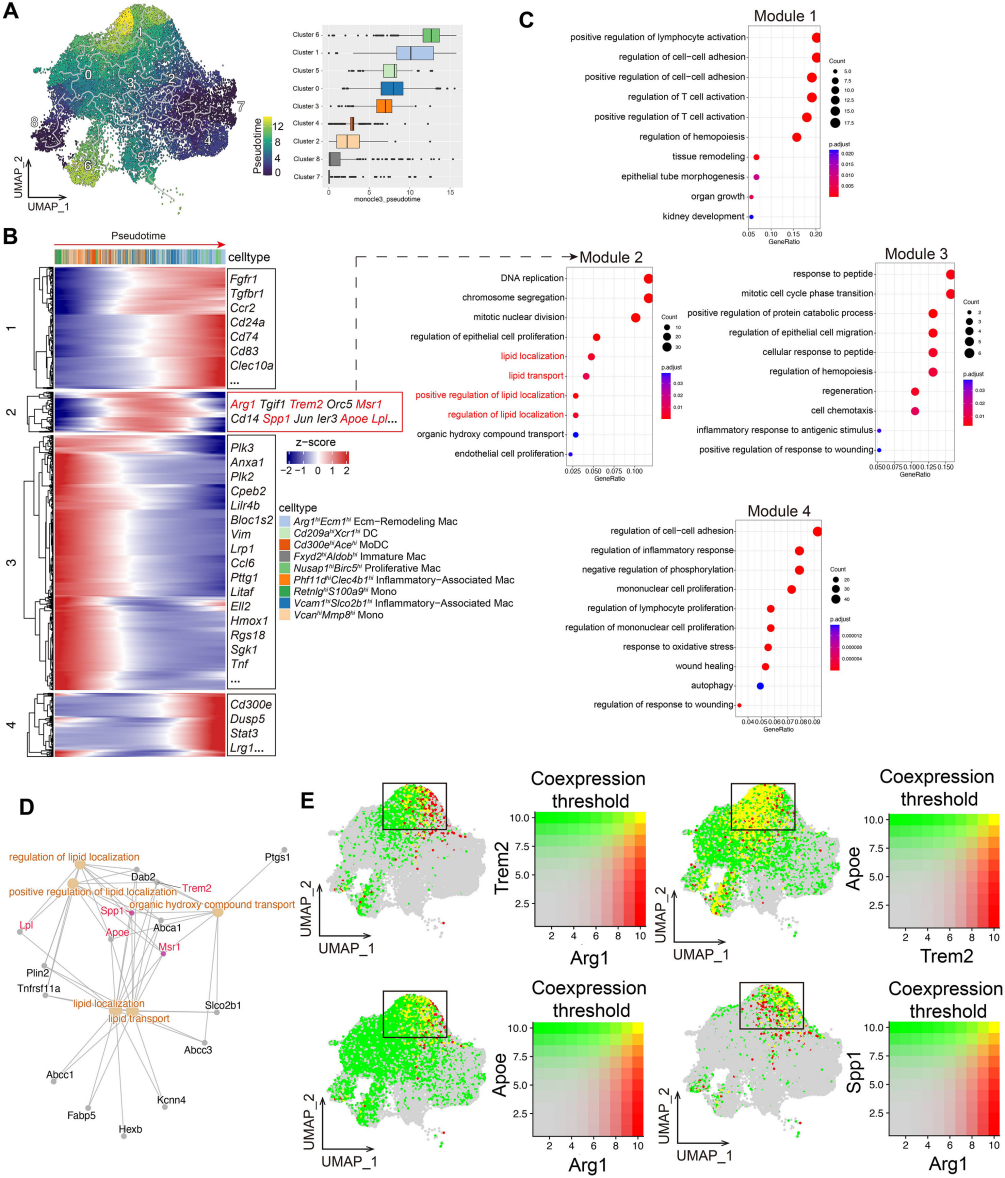
Trem2 and Apoe coordinate lipid metabolic pathways in *Arg1^hi^Ecm1^hi^* ECM-Remodeling Macrophages after IRI. **(A)** Trajectory analysis positions *Arg1^hi^Ecm1^hi^* ECM-Remodeling Mac (Cluster 1) and *Nusap1^hi^Birc5^hi^* Proliferative Mac (Cluster 6) at terminal branches, indicating terminal differentiation states. **(B, C)** K-means clustering of pseudotime-dependent genes identifies four dynamic modules. Module 1 is enriched in late-stage lymphocyte activation and tissue remodeling, Module 2 in intermediate lipid metabolism (including lipid localization and transport), Module 3 in early injury responses (hematopoiesis, regeneration, chemotaxis), and Module 4 in adhesion and inflammatory regulation. *Trem2*, *Arg1*, *Spp1*, *Msr1*, *Apoe*, and *Lpl* cluster within Module 2. **(D)** *Trem2*, *Msr1*, *Lpl*, *Spp1*, and *Apoe* participate in lipid-related pathways. **(E)** Gene co-expression networks reveal *Trem2*, *Apoe*, and *Spp1* co-expressed with *Arg1* in *Arg1^hi^Ecm1^hi^* ECM-Remodeling Mac.

By constructing the differentiation trajectory of monocyte/macrophage subsets, we found that Cluster 1 (*Arg1^hi^Ecm1^hi^* ECM-Remodeling Mac) and Cluster 6 (*Nusap1^hi^Birc5^hi^* Proliferative Mac) are located at the terminal branches of the trajectory, suggesting that these two populations are in the final stages of macrophage differentiation (**Figure 3A**). Among them, *Arg1^hi^Ecm1^hi^* ECM-Remodeling Mac likely participate in the repair of injured renal tubules after IRI as a terminally differentiated subset. Based on single-cell pseudotime analysis, we further employed the K-means clustering algorithm to identify patterns of pseudotime-dependent genes, systematically analyzing the cooperative regulatory mechanisms and dynamic evolution of gene expression networks during the differentiation of monocytes into different macrophage subsets. Although the *Arg1^hi^Ecm1^hi^* ECM-Remodeling Mac represented a terminal differentiation state, *Trem2*, *Arg1*, *Spp1*, *Msr1*, *Apoe* and *Lpl* were most prominently and coordinately activated at intermediate pseudotime, clustering in Module 2 (**Figure 3B**). We then performed functional enrichment analysis for the four modules. The results revealed that genes in Module 1 are predominantly upregulated in the terminal stages of differentiation and are enriched in pathways such as positive regulation of lymphocyte activation and tissue remodeling. Genes in Module 2 are mainly upregulated in the intermediate stages and involve multiple lipid metabolism-related pathways, including DNA replication, regulation of epithelial cell proliferation, lipid localization, lipid transport, positive regulation of lipid localization, and regulation of lipid localization. Module 3 corresponds to the early stages of differentiation, with rapid responses to injury and enrichment in pathways such as regulation of hemopoiesis, regeneration, and cell chemotaxis. Module 4 is mainly involved in regulation of cell-cell adhesion and regulation of inflammatory response, suggesting its basic role in maintaining renal tubular homeostasis following IRI (**Figure 3C**). Further analysis showed that *Trem2*, *Msr1*, *Lpl*, *Spp1*, and *Apoe* primarily participate in lipid-related pathways such as lipid localization, lipid transport, positive regulation of lipid localization and regulation of lipid localization (**Figures 3C, D**). Among these, *Apoe* and *Lpl* are expressed in multiple macrophage subsets, lacking specificity (**Supplementary Figures 2A, B**). Notably, the DEGs of *Arg1^hi^Ecm1^hi^* ECM-Remodeling Mac were also enriched in lipid localization (**Figure 1G**), suggesting that its pro–tissue repair function may be associated with lipid metabolism. Gene co-expression analysis indicated that *Trem2*, *Apoe*, and *Spp1* are co-expressed with *Arg1*, and *Apoe* is also co-expressed with *Trem2* in *Arg1^hi^Ecm1^hi^* ECM-Remodeling Mac (**Figure 3E**). This co-expression pattern was barely detectable in the control group, but it was activated on days 1, 3, 7, and 14 post-IRI (**Supplementary Figure 2C**). It is noteworthy that tubular debris in the kidney after IRI is rich in lipid components, which can be cleared by activating the *Trem2* receptor. *Trem2* is specifically expressed in the monocyte/macrophage subsets (**Supplementary Figure 2D**, **Figure 2C**), and its expression in *Arg1^hi^Ecm1^hi^* ECM-Remodeling Mac peaks on day 3 post-IRI (**Supplementary Figure 2E**), consistent with the peak of this subset in the renal interstitium at day 3 (**Supplementary Figure 1C**).

Based on these findings, we further examined Apoe signaling interactions among cell subsets after IRI. Apoe signaling was markedly activated in *Arg1^hi^Ecm1^hi^* ECM-Remodeling macrophages at days 1, 2, 3, 4, 7, 11, and 14 post-IRI, whereas no significant activation was observed in this subset in control kidneys (**Supplementary Figure 3A**). This pathway primarily mediates intercellular communication through interactions between Apoe and its receptors Trem2. Among macrophage subsets, Apoe–Trem2 interactions were most prominent in *Arg1^hi^Ecm1^hi^* ECM-Remodeling macrophages (Cluster 1) (**Supplementary Figure 3B**).

Previous studies have shown that Trem2 mediates the recognition of injury-related lipids and, as a co-stimulatory molecule, maintains microglial survival and promotes their proliferation (14). Therefore, we speculate that Trem2 not only plays a key role in lipid homeostasis and debris clearance but may also support the survival of *Arg1^hi^Ecm1^hi^* ECM-Remodeling Mac, thereby playing a critical role in the repair of injured renal tubular epithelial cells.

### 3.4 Trem2 is co-expressed with Arg1 in renal macrophages during early IRI, and its inhibition exacerbates kidney injury

**Figure 4:**
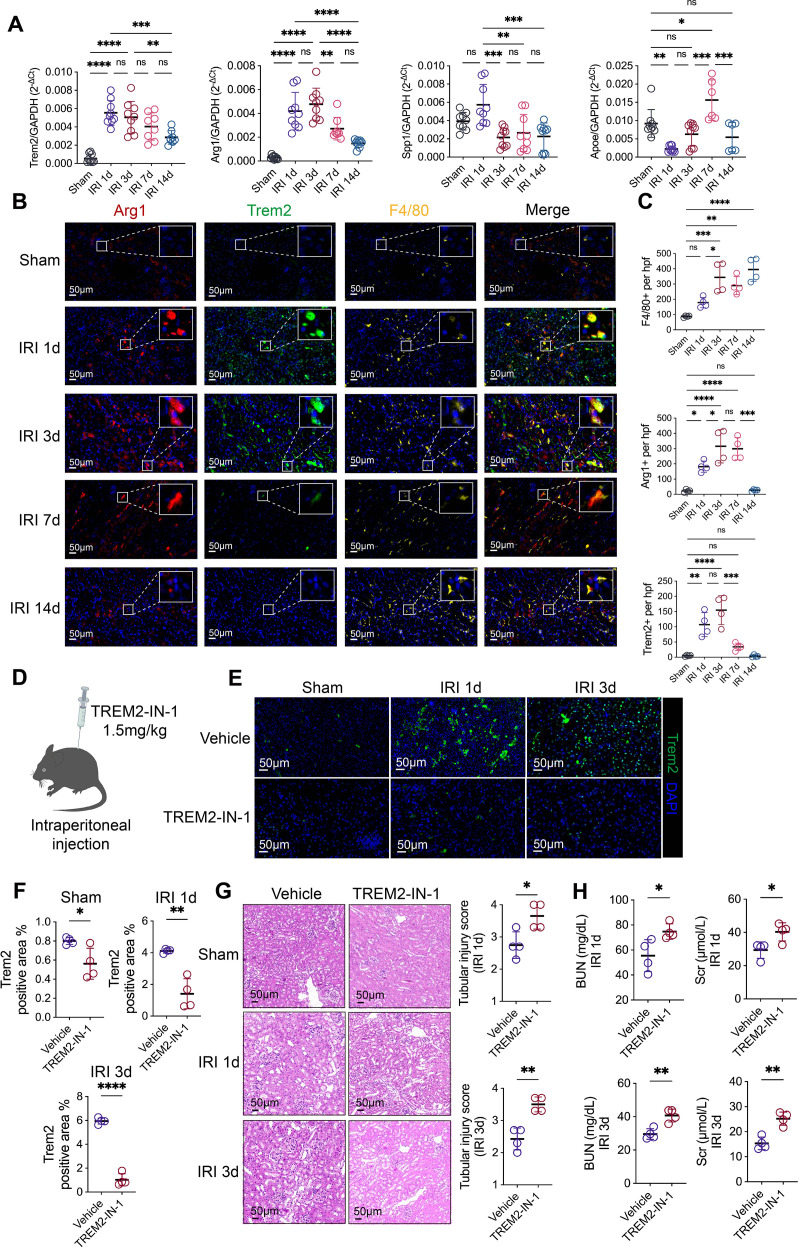
Trem2 is co-expressed with Arg1 in renal macrophages during early IRI, and its inhibition exacerbates acute kidney injury. **(A)** Dynamic expression profiles of *Arg1*, *Trem2*, *Spp1*, and *Apoe* mRNA levels in kidney tissues at various time points post-IRI (*n* = 8-9). **(B, C)** Multiplex immunofluorescence staining and quantitative analysis illustrating the spatial distribution and infiltration of Trem2^+^, Arg1^+^, and F4/80^+^ cells in sham-operated kidneys and at 1, 3, 7, and 14 days post-IRI (*n* = 4). **(D)** Schematic illustration of the experimental protocol: mice received intraperitoneal injections of the Trem2 inhibitor (TREM2-IN-1) prior to IRI induction. **(E, F)** Immunofluorescence analysis and quantification demonstrating a significant downregulation of Trem2 protein expression in the TREM2-IN-1–treated group compared to the control group at days 1 and 3 post-IRI (*n* = 4). **(G)** Histopathological evaluation of the kidney showing that pharmacological inhibition of Trem2 significantly aggravates tubular epithelial injury following IRI. **(H)** Kidney function assessment (serum BUN and Scr levels) confirming that TREM2-IN-1 treatment further worsens renal impairment during the early phase of IRI (days 1 and 3) (*n* = 4). Significance was evaluated using Student’s unpaired *t* test and one-way ANOVA followed by Tukey’s test. Tubular injury score were analyzed using the Kruskal–Wallis test followed by Dunn’s multiple comparisons test. ^*^P < 0.05, ^**^P < 0.01, ^***^P < 0.001, ^****^P < 0.0001, *ns*, no significance.

We established ischemia–reperfusion injury (IRI) model and collected serum samples at multiple time points post-IRI (days 1, 2, 3, 4, 7, 11, and 14) to dynamically assess kidney function (**Supplementary Figure 4A**). Serum BUN and Scr levels markedly increased on day 1 post-IRI, followed by an improvement starting on day 2, indicating the initiation of tubular repair. Kidney function showed substantial recovery on day 3, suggesting peak regenerative activity. Although BUN and Scr levels gradually returned toward baseline, they increased again by day 14, potentially reflecting irreversible injury and progression toward chronic kidney disease (**Supplementary Figure 4B**). We next evaluated the renal transcriptional levels of *Arg1*, *Trem2*, *Spp1*, and *Apoe* after IRI. *Trem2* and *Arg1* mRNA were significantly upregulated on days 1 and 3 post-IRI and exhibited similar expression patterns. *Spp1* expression peaked on day 1 and gradually declined, whereas *Apoe* expression increased starting on day 7 (**Figure 4A**). These results indicate that *Trem2* and *Arg1* upregulation coincides with the peak of tubular injury following IRI. Multiplex immunofluorescence staining showed that F4/80^+^ macrophages, Trem2^+^ cells, and Arg1^+^ cells increased as early as day 1 post-IRI and peaked on day 3 (**Figures 4B, C**). Co-localization analysis revealed significantly higher Pearson’s correlation and Manders’ overlap coefficients for Trem2 and Arg1 on days 1, 3, and 7 compared with controls, confirming their spatial co-expression. This co-localization weakened by day 14 (**Supplementary Figures 4C, D**).

However, immunofluorescence identified Trem2 expression beyond the Arg1^+^ lineage, with a subset of Arg1^-^ myeloid cells also exhibiting Trem2 positivity in IRI kidneys. This suggests that Trem2 may exert broader regulatory effects across the renal myeloid niche, rather than being restricted to reparative macrophages. Future studies employing flow cytometry and functional assays are required to define the specific contribution of this Trem2^+^ Arg1^-^ population to IRI progression.

Given the partial overlap in surface markers between dendritic cells (DCs) and macrophages within the renal microenvironment, we performed double immunofluorescence staining to exclude potential interference from DCs and precisely define the identity of Trem2^+^ cells. The results demonstrated that while the infiltration of Cd11c**^+^** DCs was significantly elevated in the kidney following IRI compared to the sham group, these Cd11c^+^ cells exhibited no detectable Trem2 expression. These findings effectively rule out the possibility that Trem2^+^ cells represent dendritic cells, further substantiating that Trem2 is primarily and specifically expressed in the macrophage population within the injured kidney (**Supplementary Figures 4E–G**).

To further investigate the role of Trem2 in early renal repair following IRI, the Trem2 inhibitor TREM2-IN-1 was administered intraperitoneally immediately before IRI induction (**Figure 4D**). Immunofluorescence analysis showed that Trem2 protein expression in the TREM2-IN-1 treated kidney was markedly reduced at days 1 and 3 after IRI compared with controls (**Figures 4E, F**). Concomitantly, tubular epithelial injury was significantly aggravated (**Figure 4G**), and kidney function was further impaired in the TREM2-IN-1–treated group (**Figure 4H**). Given that Arg1^+^ macrophages promote tubular regeneration through the release of soluble factors (8) and that Trem2 is co-expressed with Arg1 in renal macrophages during the early phase of IRI, we propose that Trem2 facilitates renal repair by regulating the biological functions of Arg1^+^ macrophages.

### 3.5 Tubular cell debris induces Trem2 upregulation and enhances proliferative activity in Arg1^+^ macrophages

**Figure 5:**
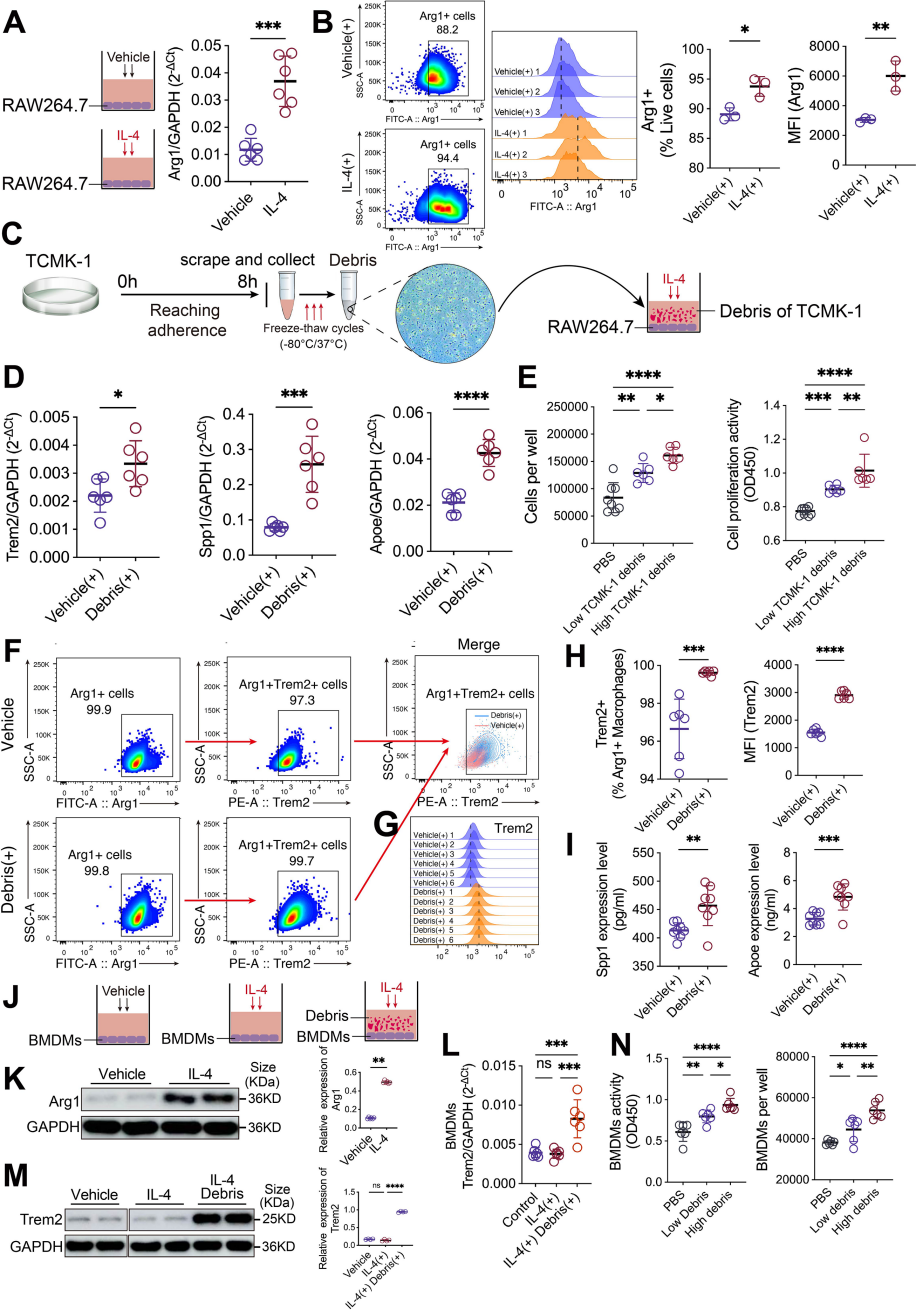
Tubular cell debris triggers Trem2 upregulation and stimulates proliferation in Arg1^+^ macrophages. **(A, B)** IL-4 treatment of RAW264.7 cells for 24 h significantly increased Arg1 transcription, Arg1^+^ macrophage proportion, and intracellular Arg1 protein intensity (*n* = 3-6). **(C, D)** To mimic the IRI microenvironment, freeze-thaw–induced tubular cell debris were co-cultured with IL-4–pretreated RAW264.7 cells (*n* = 6). This induced robust upregulation of *Trem2*, *Spp1*, and *Apoe* transcripts. **(E)** Tubular cell debris increased both the number and proliferative activity of Arg1^+^ macrophages. Higher debris concentrations further increased both measures, suggesting proliferation scales with debris exposure (*n* = 6). **(F, G, H)** Flow cytometry revealed increased Trem2 receptor intensity on Arg1^+^ macrophages and a higher proportion of Trem2^+^Arg1^+^ macrophages after debris stimulation (*n* = 6). **(I)** Levels of Spp1 and Apoe in culture supernatants were significantly elevated following debris treatment (*n* = 8). **(J)** Schematic illustration of the experimental design. Mouse primary BMDMs were pretreated with IL-4 to induce differentiation toward an Arg1^high^ phenotype, followed by co-culture with renal tubular debris. **(K)** Western blot analysis showed that IL-4 stimulation markedly upregulated Arg1 protein expression in BMDMs (*n* = 6). **(L, M)** RT-qPCR and Western blot analyses confirmed that renal tubular debris further induced the transcriptional and translational upregulation of Trem2 in Arg1^high^ BMDMs (*n* = 4). **(N)** Renal tubular debris promoted the viability and proliferation of Arg1^high^ BMDMs in a concentration-dependent manner (*n* = 6). Significance was evaluated using Student’s unpaired *t* test and one-way ANOVA followed by Tukey’s test. ^*^P < 0.05, ^**^P < 0.01, ^***^P < 0.001, ^****^P < 0.0001, *ns*, no significance.

IL-4 has been shown to significantly induce the polarization of macrophages to the M2 phenotype (47). After treating RAW264.7 cells with IL-4 for 24 hours, we observed a significant upregulation of Arg1 transcription (**Figure 5A**). Flow cytometry analysis further revealed that IL-4 treatment significantly increased the proportion of Arg1^+^ macrophages and enhanced the average fluorescence intensity of Arg1 protein within the cells (**Figure 5B**, **Supplementary Figure 5A**). To mimic the tubular microenvironment during IRI, we subjected TCMK-1 cells to freeze-thaw cycles to prepare tubular cell debris, which were co-cultured with IL-4-pretreated RAW264.7 cells for 24 hours (**Figure 5C**). The results showed that co-culturing induced significant upregulation of the transcription levels of Trem2, Spp1, and Apoe (**Figure 5D**). We next investigated the effect of low and high concentrations of tubular cell debris on the proliferative activity of Arg1^+^ macrophages. The results demonstrated that treatment with tubular cell debris led to a significant increase in the number of Arg1^+^ macrophages and enhanced their proliferative activity. At higher concentrations of tubular cell debris, both RAW264.7 cell numbers and proliferative activity further increased relative to the lower-concentration group, indicating a debris dose-dependent enhancement of Arg1^+^ macrophage proliferation (**Figure 5E**). Flow cytometric analysis showed that treatment with tubular cell debris significantly increased the average fluorescence intensity of the Trem2 receptor on Arg1^+^ macrophages and enhanced the proportion of Trem2^+^Arg1^+^ macrophages (**Figures 5F-H**, **Supplementary Figure 5B**). Additionally, we observed that the levels of Spp1 and Apoe in the Arg1^+^ macrophages supernatant from the debris-treated group were significantly higher than those in the control group (**Figure 5I**).

Next, primary mouse bone marrow–derived macrophages (BMDMs) were pretreated with IL-4 to induce differentiation into Arg1^high^ BMDMs, followed by co-culture with tubular cell debris (**Figure 5J**). Western blot analysis showed that IL-4 stimulation significantly upregulated Arg1 expression in BMDMs (**Figure 5K**, **Supplementary Figure 6A**). On this basis, treatment with tubular cell debris further markedly increased Trem2 expression at both the transcriptional and protein levels (**Figures 5L, M**, **Supplementary Figure 6B**). Finally, IL-4–pretreated BMDMs were stimulated with increasing concentrations of tubular cell debris, and cell viability and cell number were assessed. The results demonstrated that tubular cell debris significantly enhanced the viability and abundance of Arg1^high^ BMDMs in a concentration-dependent manner (**Figure 5N**).

In summary, these *in vitro* experiments further validate our previous single-cell analysis results, confirming that tubular cell debris stimulates Arg1^+^ macrophages to upregulate Trem2 expression. This is accompanied by increased paracrine signaling of Apoe and Spp1, and enhanced proliferative activity in Arg1^+^ macrophages.

### 3.6 The survival and repair function of Arg1^+^ macrophages depend on Trem2 expression

**Figure 6:**
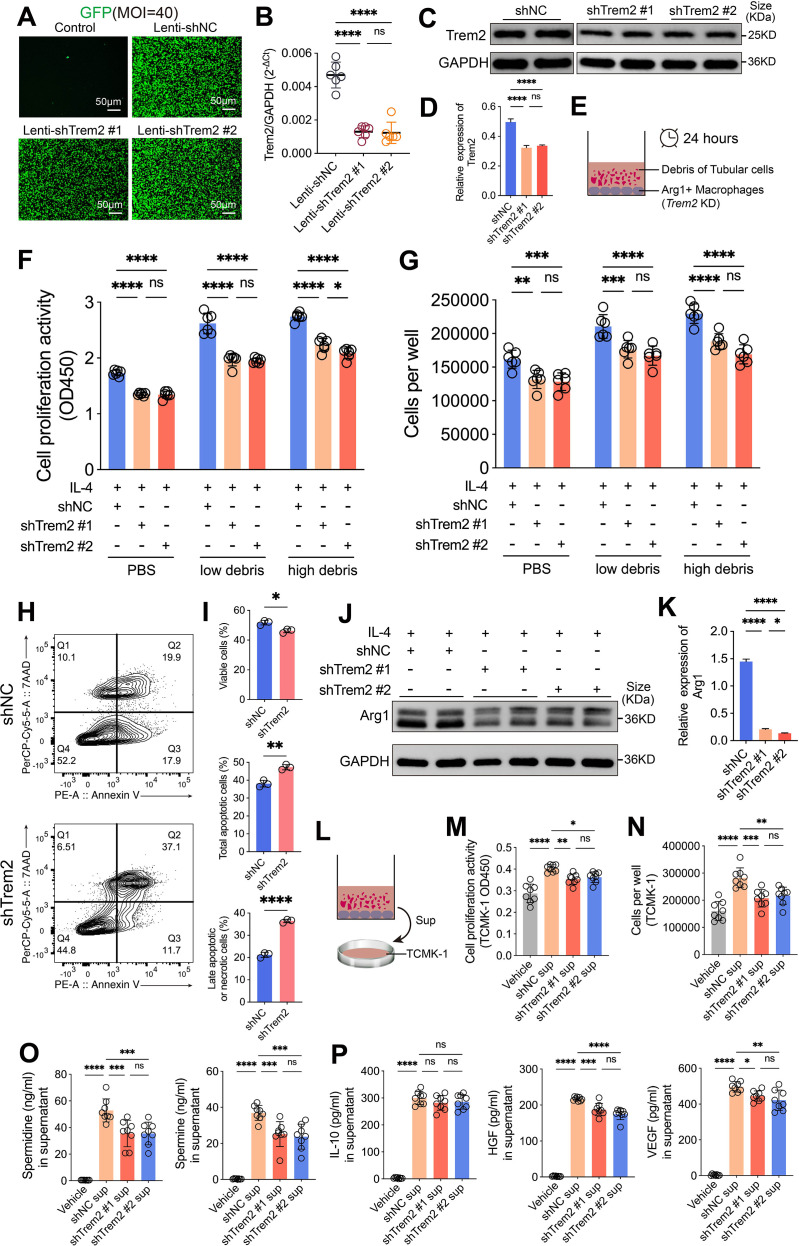
Trem2 is essential for the survival and repair function of Arg1^+^ macrophages. **(A–D)** Establishment of stable Trem2 knockdown (KD) RAW264.7 cells using shRNA lentiviral transduction, confirmed by GFP fluorescence, qPCR, and Western blot (*n* = 3-6). **(E)** Schematic illustration of the co-culture system of IL-4–pretreated Trem2 KD RAW264.7 cells with tubular cell debris. **(F)** Trem2 deficiency markedly impaired debris-induced proliferation of Arg1^+^ macrophages across both low and high debris concentrations (*n* = 6). **(G)** Debris-induced expansion of Arg1^+^ macrophages was significantly reduced upon Trem2 Knockdown (*n* = 6). **(H, I)** Apoptosis assays showed increased apoptosis of Arg1^+^ macrophages under Trem2 Knockdown (*n* = 3). **(J, K)** Trem2 Knockdown attenuated IL-4–induced Arg1 expression, suggesting impaired polarization toward a pro-repair phenotype (*n* = 3-6). **(L–N)** Conditioned medium from Trem2-sufficient Arg1^+^ macrophages promoted TCMK-1 cell proliferation and expansion, whereas Trem2 KD abolished this pro-regenerative effect (*n* = 8). **(O)** Levels of spermidine and spermine in the supernatants of Arg1^+^ macrophages were increased upon stimulation with tubular debris and reduced by Trem2 knockdown (*n* = 8). **(P)** Tubular debris stimulation increased HGF and VEGF levels in Arg1^+^ macrophage supernatants, which were reduced by Trem2 knockdown, while IL-10 levels remained unchanged (*n* = 8). Significance was evaluated using Student’s unpaired *t* test, one-way ANOVA, or two-way ANOVA followed by Tukey’s test. ^*^P < 0.05, ^**^P < 0.01, ^***^P < 0.001, ^****^P < 0.0001, *ns*, no significance.

To further investigate the role of Trem2, we constructed stable *Trem2* knockdown RAW264.7 cell lines using shRNA. These cells were transduced with lentiviral vectors and selected with puromycin, successfully achieving Trem2 Knockdown. GFP fluorescence was successfully expressed in RAW264.7 cells following lentiviral transduction (**Figure 6A**), and the knockdown efficiency of *Trem2* exceeded 70% (**Figure 6B**). Western blot analysis further confirmed the stable Knockdown of Trem2 protein in RAW264.7 cells (**Figures 6C, D**, **Supplementary Figure 6C**). Subsequently, we pre-treated Trem2 Knockdown RAW264.7 cells with IL-4 and co-cultured them with renal tubular cell debris for 24 hours (**Figure 6E**). The results showed that stimulation with debris significantly enhanced the proliferation of Arg1^+^ macrophages. Compared to the low-concentration debris group, the high-concentration group further promoted the proliferation activity of Arg1^+^ macrophages. However, in the Trem2 Knockdown condition, both the low- and high-concentration renal tubular cell debris groups exhibited a marked reduction in Arg1^+^ macrophage proliferation (**Figure 6F**). Furthermore, the changes in Arg1^+^ macrophage numbers were consistent with the cell proliferation assay results. Stimulation with renal tubular epithelial cell debris significantly increased the number of Arg1+ macrophages, but this increase was significantly reversed upon Trem2 Knockdown (**Figure 6G**). Additional apoptosis assays revealed that in the Trem2 Knockdown condition, apoptosis of Arg1^+^ macrophages was significantly increased (**Figures 6H, I**), suggesting that Trem2 plays a crucial role in maintaining the survival of Arg1^+^ macrophages under renal tubular epithelial cell debris stimulation. Moreover, we explored whether Trem2 Knockdown would affect the expression of Arg1 in RAW264.7 cells following IL-4 stimulation. The results showed that Trem2 Knockdown weakened the macrophage response to IL-4 stimulation, with a significant reduction in Arg1 expression (**Figures 6J, K**, **Supplementary Figure 6D**). This indicates that macrophages rely on Trem2 expression to undergo phenotype conversion to a pro-repair state in response to IL-4. Previous studies have shown that Arg1^+^ macrophages promote renal tubular regeneration and repair during AKI by secreting soluble pro-regenerative factors (8). To further verify whether Trem2 Knockdown affects the repair function of Arg1^+^ macrophages, we added the culture supernatant of Trem2 Knockdown Arg1^+^ macrophages to TCMK-1 cell cultures to assess its effect on TCMK-1 cell proliferation (**Figure 6L**). The results showed that the culture supernatant from the shNC transfected group significantly promoted TCMK-1 cell proliferation, whereas the supernatant from the Trem2 Knockdown group notably inhibited TCMK-1 proliferation (**Figure 6M**). Similarly, the culture supernatant from the shNC group significantly increased the number of TCMK-1 cells, while the Trem2 Knockdown group reduced the number of TCMK-1 cells significantly (**Figure 6N**). We further examined the soluble factors related to cell proliferation in the supernatants of Arg1^+^ macrophages. Arg1 provides precursors for polyamine synthesis through its catalytic activity. The results revealed that the polyamines spermidine and spermine—both crucial mediators of cellular proliferation and differentiation (48)—were markedly elevated upon stimulation with debris. Notably, their levels were significantly reduced following Trem2 knockdown (**Figure 6O**). In parallel, we assessed the levels of the classical paracrine factors IL-10, HGF, and VEGF in the supernatants. IL-10, HGF, and VEGF were all markedly increased; however, upon Trem2 knockdown, HGF and VEGF levels declined significantly, whereas IL-10 remained unchanged (**Figure 6P**). Collectively, these findings indicate that tubular cell debris activates Trem2 to sustain the survival of Arg1^+^ macrophages, thereby promoting the secretion of polyamines, HGF, and VEGF—factors that, in turn, enhance tubular epithelial regeneration and repair.

### 3.7 Trem2 inhibition compromises Arg1^high^ BMDM survival and their pro-proliferative effects on tubular cells

**Figure 7:**
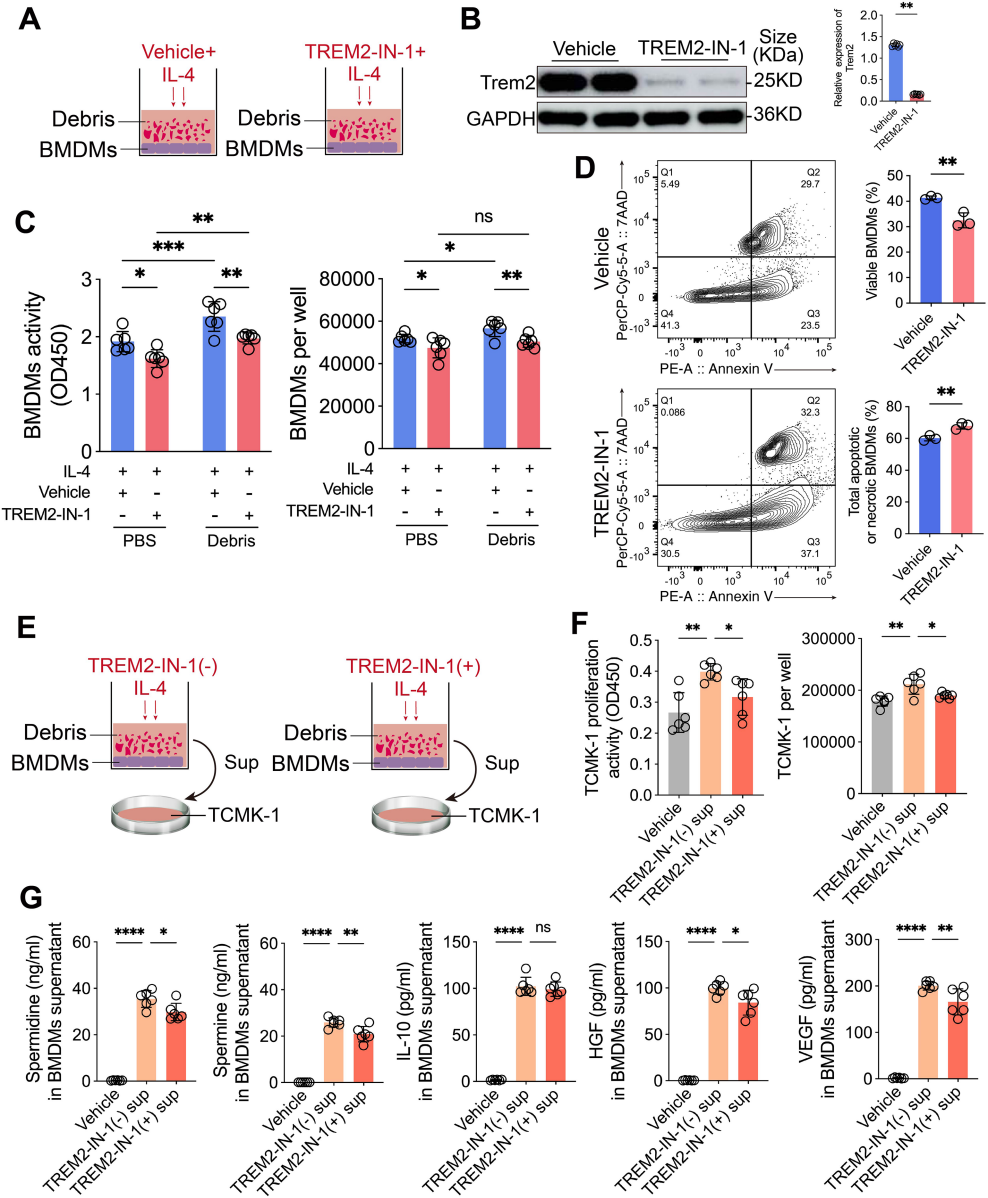
Inhibition of Trem2 reduces the survival of Arg1^high^ BMDMs and impairs their ability to promote renal tubular epithelial cell proliferation. **(A)** Schematic illustration of the experimental design: BMDMs were treated with TREM2-IN-1 in combination with IL-4 and subsequently co-cultured with renal tubular cell debris. **(B)** Western blot analysis confirmed that TREM2-IN-1 markedly downregulated Trem2 protein levels in Arg1^high^ BMDMs (*n* = 6). **(C)** Tubular cell debris enhanced the viability of Arg1^high^ BMDMs, whereas TREM2-IN-1 treatment significantly reduced both BMDMs viability and BMDMs number (*n* = 6). **(D)** Flow cytometry revealed that inhibition of Trem2 significantly decreased the survival rate of Arg1^high^ BMDMs and markedly increased apoptosis (*n* = 3). **(E)** Schematic of the conditioned medium experiment: Culture supernatants were collected from Arg1^high^ BMDMs and applied to TCMK-1 cells. **(F)** Conditioned medium from BMDMs treated with IL-4 and renal tubular cell debris significantly promoted TCMK-1 cell proliferation, whereas the addition of TREM2-IN-1 markedly attenuated this pro-proliferative effect (*n* = 6). **(G)** Measurement of Arg1^high^ BMDM-derived secreted factors: TREM2-IN-1 treatment significantly reduced the levels of spermidine, spermine, HGF, and VEGF in the conditioned medium, while IL-10 levels remained unchanged (*n* = 6). Significance was evaluated using Student’s unpaired *t* test, one-way ANOVA, or two-way ANOVA followed by Tukey’s test. ^*^P < 0.05, ^**^P < 0.01, ^***^P < 0.001, ^****^P < 0.0001, *ns*, no significance.

To further validate the role of Trem2 expression in maintaining the survival of Arg1^+^ macrophages, BMDMs were treated with IL-4 in the presence or absence of the Trem2 inhibitor TREM2-IN-1 and subsequently co-cultured with tubular cell debris (**Figure 7A**). Western blot analysis showed that TREM2-IN-1 treatment significantly reduced Trem2 protein expression in BMDMs (**Figure 7B**, **Supplementary Figure 6E**). Tubular cell debris markedly enhanced the viability and abundance of Arg1^high^ BMDMs; however, upon TREM2-IN-1 treatment, both cell viability and cell number of Arg1^high^ BMDMs were significantly decreased (**Figure 7C**). Flow cytometric analysis further demonstrated that inhibition of Trem2 significantly reduced the survival proportion of Arg1^high^ BMDMs, accompanied by a marked increase in overall apoptosis (**Figure 7D**). Next, culture supernatants from BMDMs were collected and applied to TCMK-1 cells (**Figure 7E**). Supernatants derived from BMDMs treated with IL-4 in combination with tubular cell debris significantly promoted TCMK-1 cell proliferation and cell number; in contrast, supernatants from BMDMs additionally treated with TREM2-IN-1 exhibited a markedly attenuated pro-proliferative effect on TCMK-1 cells (**Figure 7F**). Finally, the concentrations of spermidine, spermine, IL-10, HGF, and VEGF in the BMDM culture supernatants were quantified. Compared with controls, levels of spermidine, spermine, HGF, and VEGF were significantly reduced in supernatants from the TREM2-IN-1–treated group, whereas IL-10 levels showed no significant difference (**Figure 7G**). These findings are consistent with our previous observations.

### 3.8 Knockdown or inhibition of Trem2 decreases the survival of Arg1^+^ macrophages via Pten activation and Bcl2 suppression

**Figure 8:**
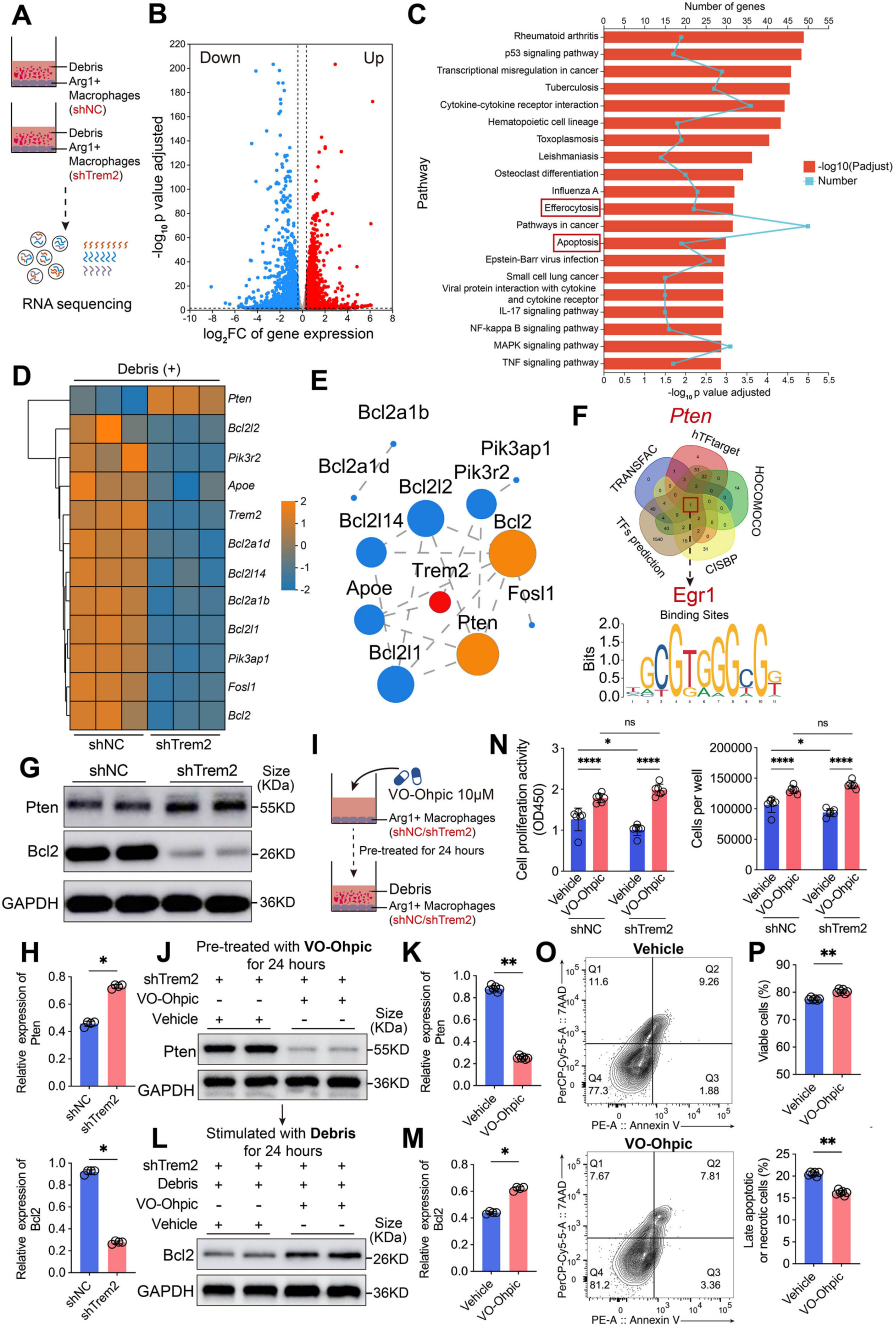
Knockdown of Trem2 decreases the survival of Arg1^+^ macrophages via Pten activation and Bcl2 suppression. **(A, B)** RNA-seq comparing control and Trem2 knockdown Arg1^+^ macrophages co-cultured with tubular cell debris identified DEGs; FDR < 0.05, |Fold Change| > 1.5). **(C)** KEGG pathway enrichment revealed significant changes in efferocytosis and apoptosis pathways, indicating impaired debris clearance and altered apoptotic regulation upon Trem2 Knowdown. **(D)** Trem2 Knockdown downregulated anti-apoptotic proteins *Bcl2* downstream of PI3K-AKT while upregulating the negative regulator *Pten*. **(E)** Protein-protein interaction network showed Pten links to multiple anti-apoptotic proteins. **(F)** Transcription factor analysis predicted Egr1 as a potential upstream regulator of *Pten*. **(G, H)** Western blot showing increased Pten and decreased Bcl2 expression in Trem2-knockdown Arg1^+^ macrophages (*n* = 4). **(I)** Experimental design: macrophages were pretreated with VO-Ohpic (Pten inhibitor) and co-cultured with renal tubular debris. **(J, K)** VO-Ohpic treatment significantly inhibited Pten expression in Trem2-knockdown Arg1^+^ macrophages (*n* = 6). **(L, M)** Under co-culture conditions, Bcl2 protein expression was restored in VO-Ohpic-treated cells (*n* = 4). **(N)** VO-Ohpic treatment rescued proliferative activity and increased cell numbers in Trem2-knockdown Arg1^+^ macrophages compared with the vehicle group (*n* = 6). **(O, P)** VO-Ohpic reduced late apoptosis and necrosis, and improved viability of Trem2-knockdown Arg1^+^ macrophages (*n* = 6). Significance was evaluated using Student’s unpaired *t* test, one-way ANOVA, or two-way ANOVA followed by Tukey’s test and Mann–Whitney U test for non-parametric comparisons. ^*^P < 0.05, ^**^P < 0.01, ^***^P < 0.001, ^****^P < 0.0001, *ns*, no significance.

To further explore the impact of renal tubular epithelial cell debris on the transcriptional profile of Arg1^+^ macrophages following Trem2 knockdown, we performed RNA sequencing (**Figure 8A**). DEGs were identified using criteria of false discovery rate (FDR) < 0.05 and |Fold Change| > 1.5 (**Figure 8B**). KEGG pathway enrichment analysis was then performed to identify the biological functions potentially impacted by these DEGs. Notably, the Efferocytosis and Apoptosis pathways were among the top 20 enriched pathways (**Figure 8C**), confirming that Trem2 knockdown significantly impaired the debris clearance ability of Arg1^+^ macrophages, while also altering their regulation of apoptosis. We observed that, following Trem2 knockdown, the expression of anti-apoptotic proteins Bcl2, which are downstream of the PI3K-AKT signaling pathway, was markedly downregulated. In contrast, the expression of the negative regulator of the PI3K-AKT pathway, Pten, was significantly upregulated (**Figure 8D**). The protein interaction network analysis revealed that Pten interacts with several downstream anti-apoptotic proteins (**Figure 8E**). Further investigation of upregulated transcription factors from the DEGs, in combination with multiple databases, predicted that Egr1 may regulate *Pten* expression (**Figure 8F**). Western blot analysis showed that, compared with the control group, Pten protein expression was markedly upregulated, while Bcl2 protein expression was significantly downregulated in Trem2-knockdown Arg1^+^ macrophages (**Figures 8G, H**, **Supplementary Figure 7A**). This result was consistent with the RNA-seq data. To further investigate whether Trem2 knockdown suppresses the downstream anti-apoptotic factor Bcl2 by upregulating Pten and thereby inhibiting the PI3K-AKT signaling pathway, we pretreated both control and Trem2-knockdown Arg1^+^ macrophages with VO-Ohpic, a specific and potent inhibitor of Pten, followed by co-culture with renal tubular debris (**Figure 8I**). The results demonstrated that VO-Ohpic markedly inhibited Pten expression in Trem2-knockdown Arg1^+^ macrophages (**Figures 8J, K**, **Supplementary Figure 7B**). Under co-culture conditions with renal tubular debris, Bcl2 protein levels were significantly elevated in the VO-Ohpic-treated group (**Figures 8L, M**, **Supplementary Figure 7C**). Compared with the vehicle group, VO-Ohpic treatment restored the proliferative activity and cell number of Trem2-knockdown Arg1^+^ macrophages (**Figure 8N**), while reducing the proportion of late apoptotic and necrotic cells and improving overall cell viability (**Figures 8O, P**).

**Figure 9:**
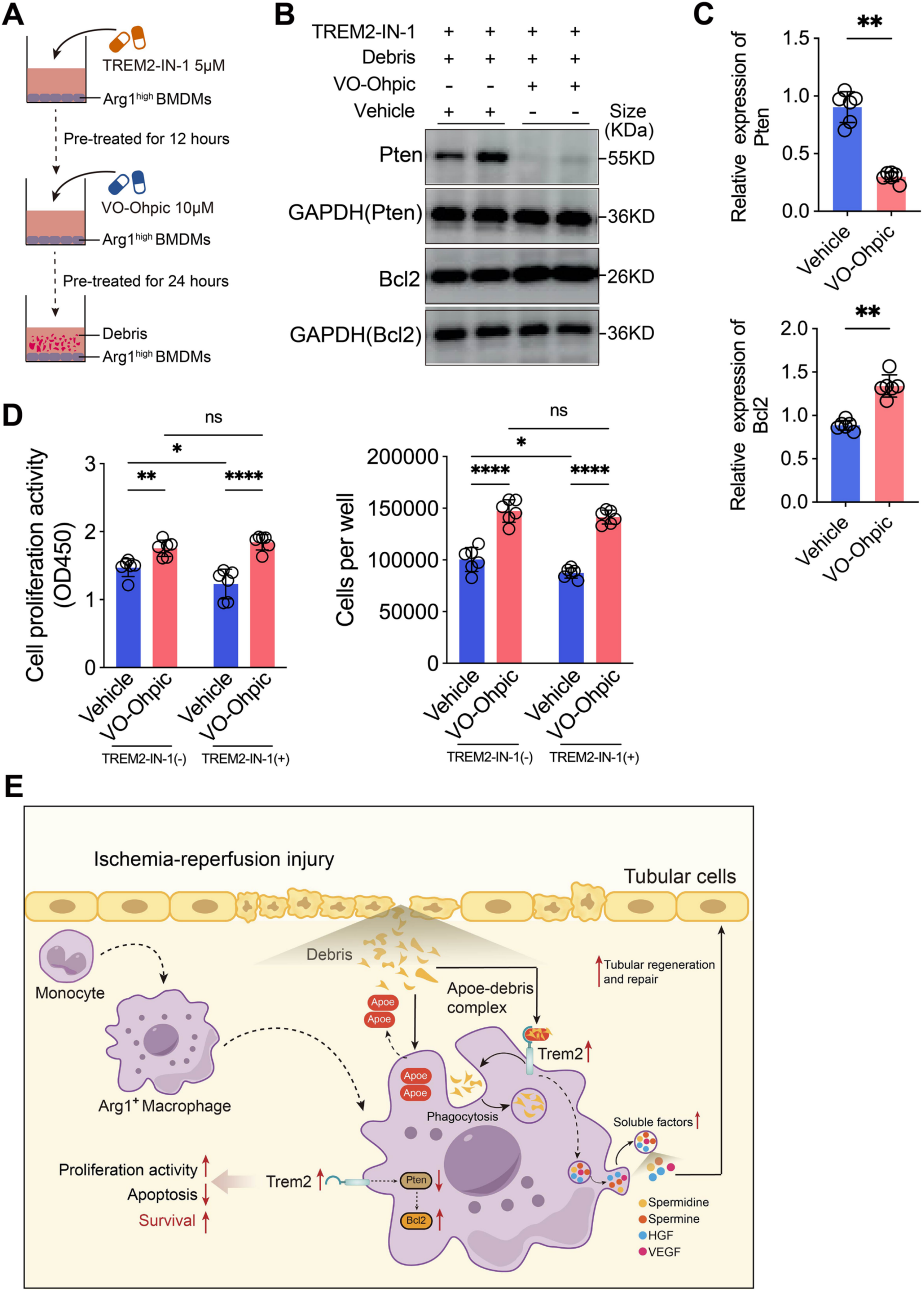
Inhibition of Ttem2 reduces the viability of Arg1^high^ BMDMs by upregulating Pten and suppressing Bcl2. **(A)** Schematic illustration of the experimental design: IL-4–induced Arg1^high^ BMDMs were first treated with TREM2-IN-1 to inhibit Trem2, followed by treatment with the PTEN inhibitor VO-Ohpic, and subsequently co-cultured with renal tubular cell debris. **(B, C)** Western blot analysis showed that, compared with the control group, VO-Ohpic treatment significantly downregulated Pten protein expression in Arg1^high^ BMDMs and markedly upregulated the expression of the key anti-apoptotic protein Bcl2 (*n* = 6). **(D)** VO-Ohpic treatment effectively reversed the TREM2-IN-1–induced reduction in cell viability and cell number of Arg1^high^ BMDMs (*n* = 6). **(E)** Proposed mechanism: Tubular cell debris generated during IRI, in conjunction with Apoe released by Arg1^+^ macrophages, activates Trem2 and further upregulates its expression. Elevated Trem2 signaling suppresses Pten and upregulates the anti-apoptotic factor Bcl2, thereby promoting debris clearance, Arg1^+^ macrophage survival. Surviving Arg1^+^ macrophages release spermidine, spermine, HGF, and VEGF, which enhance renal tubular epithelial cell regeneration and repair. Significance was evaluated using Student’s unpaired *t* test and two-way ANOVA followed by Tukey’s test. ^*^P < 0.05, ^**^P < 0.01, ^***^P < 0.001, ^****^P < 0.0001, *ns*, no significance.

Finally, Arg1^high^ BMDMs generated by IL-4 induction were treated with TREM2-IN-1, followed by administration of VO-Ohpic, and subsequently co-cultured with tubular cell debris (**Figure 9A**). Western blot analysis showed that, compared with the control group, VO-Ohpic treatment markedly reduced Pten protein expression while significantly upregulated the anti-apoptotic protein Bcl2 (**Figures 9B, C**, **Supplementary Figure 7D**). Functional assays further demonstrated that inhibition of Trem2 significantly decreased the viability and abundance of Arg1^high^ BMDMs, whereas VO-Ohpic treatment effectively reversed these effects, consistent with our previous findings (**Figure 9D**).

Based on these findings, we propose the following mechanism: during the course of IRI, renal tubular epithelial cells undergo oxidative stress-induced injury, leading to necrosis and apoptosis, and the release of epithelial cell debris. The debris, in conjunction with Apoe released by Arg1^+^ macrophages, activates the Trem2 receptor. Upon activation, Trem2 enhances the macrophages’ ability to phagocytize and clear the debris, while also upregulating Trem2 expression on the macrophages. Elevated Trem2 signaling suppresses Pten and increases the expression of the anti-apoptotic factor Bcl2, promoting cell proliferation and inhibiting apoptosis, thereby ensuring their survival. Surviving Arg1^+^ macrophages promote tubular epithelial repair by releasing spermidine, spermine, HGF, and VEGF during IRI (**Figure 9E**).

## 4 Discussion

The classical M1/M2 macrophage paradigm fails to fully capture the true phenotypic characteristics of macrophages during IRI. In this study, by integrating currently available single-cell transcriptomic datasets of renal IRI, we generated a complete atlas of renal macrophages in IRI and identified an *Arg1^hi^Ecm1^hi^* ECM-Remodeling Mac population. This subset co-expresses *Trem2*, *Spp1*, *Apoe*, and *Msr1*, is involved in lipid localization, and promotes renal tubular epithelial cell proliferation during IRI. Previous studies have considered Arg1 to be a marker of the pro-fibrotic macrophage phenotype that accelerates renal fibrosis when tubular epithelial repair is injured (49). However, existing evidence has not fully elucidated the regenerative function of Arg1^+^ macrophages in the early phase of IRI, nor their extent of renal infiltration at this stage. Using murine IRI models at different time points, we found that Arg1^+^ macrophage infiltration peaked at day 3 post-IRI and gradually declined during the late phase. Previous work has shown that early Arg1^+^ macrophages in IRI limit nitric oxide synthesis by depleting arginine, thereby attenuating inflammation, while secreting multiple pro-proliferative factors to promote tubular epithelial cell regeneration (8). Therefore, elucidating the mechanisms that sustain early Arg1^+^ macrophage survival during IRI may help preserve the regenerative capacity of the injured kidney.

The Triggering Receptor Expressed on Myeloid Cells (TREM) family is recognized as a master regulator of innate immunity and tissue homeostasis. Specifically, Trem-1, Trem-2, and the mouse-specific Trem-3 orchestrate macrophage polarization, metabolic reprogramming, and efferocytosis via an intricate signaling network (50). Among these members, Trem2 stands out as a pivotal surface receptor broadly involved in a variety of pathological processes including central nervous system diseases, cancer, metabolic syndrome, and heart disease, where it regulates key macrophage functions such as phagocytosis, migration, metabolism, lipid processing, and lysosomal degradation (50–52). Trem2 functions as a lipid sensor that recognizes danger signals by binding to its endogenous ligand, ApoE. This recognition triggers the DAP12-SYK signaling cascade, which in turn activates PI3K/Akt/mTOR-mediated metabolic reprogramming (53, 54). By modulating lipid transcription via the LXR/SREBP pathways, Trem2 ultimately drives efficient transmembrane cholesterol efflux (55). Trem2 has been shown to function as a co-stimulatory molecule that maintains microglial survival in neurodegenerative diseases (14, 56–58). Beyond the nervous system, Trem2 expression may also play a beneficial role in IRI. When tubular epithelial cells are injured, membrane lipid asymmetry is disrupted, exposing inner-leaflet lipids and generating abundant Trem2 ligands, such as phosphatidylserine (PS), phosphatidylinositol (PI), and sulfatides (Sulf), which engage Trem2 receptors and enhance the phagocytic function of Arg1^+^ macrophages. However, whether tubular epithelial cell debris modulates Trem2 expression in Arg1^+^ macrophages and whether this receptor activation is essential for their survival remain unknown.

Our study demonstrated that Trem2 expression in Arg1^+^ macrophages is upregulated in the early phase of IRI and correlates positively with the degree of tubular injury. Given that Trem2 functions as a phagocytic receptor in macrophages and is critical for clearing necrotic tissue, and inspired by its role in sustaining microglial survival, we hypothesized that Trem2 in IRI may not only mediate phagocytosis but also promote Arg1^+^ macrophage survival. Because IRI fundamentally involves mitochondrial oxidative stress–induced necrosis and apoptosis of tubular epithelial cells, resulting in abundant cellular debris, we modeled the immune microenvironment of Arg1^+^ macrophages in IRI by stimulating them with tubular epithelial cell debris. Strikingly, we found that such stimulation markedly upregulated Trem2 expression, along with the paracrine factors Spp1 and Apoe, and enhanced Arg1^+^ macrophage proliferation. Trem2 knockdown or treatment with the Trem2 inhibitor TREM2-IN-1 significantly reduced proliferative activity and increased apoptosis. These findings reveal a previously unrecognized role for Trem2 in maintaining early Arg1^+^ macrophage survival during IRI. However, the specific lipid components within tubular debris that activate Trem2 remain to be elucidated, and further studies are required to confirm this mechanism. Furthermore, during IL-4–induced reparative polarization, Arg1 expression was partially dependent on Trem2. Consistent with microglial studies, Trem2-deficient microglia display a blunted response to IL-4, with reduced STAT6 levels and markedly decreased Arg1 expression (59). This suggests that Trem2 also plays a critical role in the IL-4–induced polarization of macrophages towards a pro-repair phenotype. In our experiments, conditioned media from Arg1^+^ macrophages with Trem2 knockdown or treated with the Trem2 inhibitor TREM2-IN-1 attenuated the pro-regenerative effect on tubular cells, suggesting that these macrophages secrete soluble pro-regenerative factors. Subsequent ELISA analysis identified spermidine, spermine, HGF, and VEGF as the major mediators responsible for this effect. Collectively, Trem2 preserves Arg1^+^ macrophage survival and ensures their early pro-repair function, which is critical for tubular regeneration after IRI. This mechanism helps attenuate the progression from AKI to CKD and ultimately improves kidney function.

We next explored the downstream signaling pathways activated by Trem2 to sustain Arg1^+^ macrophage survival. Trem2 is known to form heterodimers with DNAX activation protein 12 (DAP12) and DAP10, triggering downstream signaling cascades (52). Apoe, as an apolipoprotein, is secreted by macrophages into the extracellular space, where it binds lipids and cholesterol to form lipoprotein particles. Apoe acts as lipid rafts that mediate lipid transport via endocytosis and enhance macrophage TLR signaling, thereby modulating inflammation (60). Apoe has been shown to be indispensable for Trem2-mediated phagocytosis. In addition, Trem2 sustains microglial survival through activation of the PI3K-AKT–dependent mTOR pathway (12). Trem2 may also synergize with CSF-1–CSF-1R signaling to activate spleen tyrosine kinase (Syk), which in turn triggers multiple downstream mediators such as ERK and PI3K-AKT (61), and could potentially sustain macrophage survival through Wnt/β-catenin pathway activation (56, 62). In a murine UUO model, Trem2 deletion suppressed mTOR pathway activation, thereby exacerbating kidney injury and promoting fibrosis (63). Our RNA-seq analysis of Trem2-knockdown Arg1^+^ macrophages revealed marked upregulation of *Pten*, a negative regulator of the PI3K-AKT pathway. Transcription factor prediction suggested that Egr1 may be an important upstream regulator of *Pten*. Trem2 knockdown activated the Egr1/Pten axis, thereby suppressing downstream PI3K-AKT anti-apoptotic protein Bcl2, leading to Arg1^+^ macrophage apoptosis. However, *in vitro*, treatment with the Pten inhibitor VO-Ohpic effectively reversed these effects, restoring Bcl2 expression and enhancing the survival of Arg1^+^ macrophages. In future work, we plan to investigate Egr1-mediated activation of the Pten promoter using dual-luciferase reporter assays and to further examine the effects of Trem2 deletion or overexpression on the Egr1/Pten axis and its downstream anti-apoptotic proteins.

Due to the clinical challenges of obtaining kidney biopsies during AKI and the strict ethical constraints regarding *in vivo* human manipulation, a limitation of the current study is the lack of validation for Trem2 protein expression and its functional role in human kidney samples. Although our analysis of existing human scRNA-seq datasets corroborated the transcriptional upregulation of Trem2 in myeloid cells, future prospective studies utilizing non-invasive human clinical samples are warranted to validate our key conclusion that Trem2 sustains the survival of Arg1^+^ macrophages and promotes renal repair in AKI.

Notably, Trem2 is not only expressed in its membrane-bound form but may also be cleaved to generate soluble Trem2 (sTrem2). Exogenous sTrem2 supplementation can preserve macrophage survival (64, 65), and injection of sTrem2 into infarcted mouse hearts has been shown to improve cardiac function and promote ventricular remodeling (66). Whether sTrem2 supplementation can facilitate renal repair after IRI is an intriguing question for future investigation.

Although we did not directly examine the effects of genetic Trem2 knockdown or deletion on kidney function *in vivo* after IRI, pharmacological inhibition of Trem2 using TREM2-IN-1 administered prior to IRI markedly aggravated kidney injury and further impaired kidney function in mice. These findings are consistent with previous studies demonstrating that Trem2 deficiency promotes macrophage polarization and exacerbates kidney injury in IRI models (63). Given Trem2’s central role in sustaining Arg1^+^ macrophage survival, targeted activation of Trem2 holds substantial therapeutic promise for IRI. In the field of neurodegeneration, multiple Trem2 agonists have been developed. For example, the humanized monoclonal antibody AL002 promotes microglial survival and phagocytic function and is currently in phase II clinical trials for Alzheimer’s disease (67). Recently, the Trem2-activating antibody ATV: TREM2 was shown to induce human microglial proliferation and enhance mitochondrial metabolism (68). Additionally, supplementation with cyclocreatine, a creatine analogue, can restore ATP levels in Trem2-deficient microglia and improve their survival (12). Developing small-molecule Trem2 agonists that target its active domain in Arg1^+^ macrophages, or modulating the downstream PI3K-AKT signaling pathway of Trem2 in these macrophages, may sustain their survival during the initial phase of IRI, promote renal tubular epithelial cell regeneration, and improve kidney function, thereby offering a potential novel therapeutic approach for mitigating IRI.

## Data Availability

All data supporting the conclusions of this study are available within the article and its **Supplementary Materials**. The RNA sequencing datasets generated during the present study have been deposited in the NCBI Gene Expression Omnibus (GEO) repository and are publicly accessible under the accession number **GSE306859**.

## Supplementary material

Supplementary Figure 1 Temporal dynamics and subpopulations of monocyte/macrophages during IRI.

Supplementary Figure 2 Co-expression patterns of *Arg1*, *Trem2*, *Spp1*, and *Apoe* at different time points following IRI.

Supplementary Figure 3 Activation of the Apoe-Trem2 signaling axis in *Arg1^hi^Ecm1^hi^*ECM-Remodeling Macrophages after IRI.

Supplementary Figure 4 Trem2 is upregulated early after IRI and co-localizes with Arg1 in macrophages.

Supplementary Figure 5 Representative FACS gating strategies.

Supplementary Figure 6 Original Western blot images (Trem2, Arg1).

Supplementary Figure 7 Original Western blot images (Pten, Bcl2).

## References

[1] Al-Jaghbeer M Dealmeida D Bilderback A Ambrosino R Kellum JA . Clinical decision support for in-hospital AKI. J Am Soc Nephrol. (2018) 29(2):654–60. doi: 10.1681/asn.2017070765. PMID: 29097621 PMC5791078

[2] Kellum JA Romagnani P Ashuntantang G Ronco C Zarbock A Anders HJ . Acute kidney injury. Nat Rev Dis Primers. (2021) 7(1):52. doi: 10.1038/s41572-021-00284-z. PMID: 34267223

[3] Hoste EAJ Kellum JA Selby NM Zarbock A Palevsky PM Bagshaw SM . Global epidemiology and outcomes of acute kidney injury. Nat Rev Nephrol. (2018) 14(10):607–25. doi: 10.1038/s41581-018-0052-0. PMID: 30135570

[4] Jang HR Rabb H . Immune cells in experimental acute kidney injury. Nat Rev Nephrol. (2015) 11(2):88–101. doi: 10.1038/nrneph.2014.180. PMID: 25331787

[5] Lameire NH Bagga A Cruz D De Maeseneer J Endre Z Kellum JA . Acute kidney injury: an increasing global concern. Lancet. (2013) 382(9887):170–9. doi: 10.1016/s0140-6736(13)60647-9. PMID: 23727171

[6] Chawla LS Eggers PW Star RA Kimmel PL . Acute kidney injury and chronic kidney disease as interconnected syndromes. N Engl J Med. (2014) 371(1):58–66. doi: 10.1056/NEJMra1214243. PMID: 24988558 PMC9720902

[7] Cavaillé-Coll M Bala S Velidedeoglu E Hernandez A Archdeacon P Gonzalez G . Summary of FDA workshop on ischemia reperfusion injury in kidney transplantation. Am J Transplant. (2013) 13(5):1134–48. doi: 10.1111/ajt.12210. PMID: 23566221

[8] Shin NS Marlier A Xu L Doilicho N Linberg D Guo J . Arginase-1 is required for macrophage-mediated renal tubule regeneration. J Am Soc Nephrol. (2022) 33(6):1077–86. doi: 10.1681/asn.2021121548. PMID: 35577558 PMC9161787

[9] Hoeft K Schaefer GJL Kim H Schumacher D Bleckwehl T Long Q . Platelet-instructed SPP1(+) macrophages drive myofibroblast activation in fibrosis in a CXCL4-dependent manner. Cell Rep. (2023) 42(2):112131. doi: 10.1016/j.celrep.2023.112131. PMID: 36807143 PMC9992450

[10] Zhang YL Tang TT Wang B Wen Y Feng Y Yin Q . Identification of a novel ECM remodeling macrophage subset in AKI to CKD transition by integrative spatial and single-cell analysis. Adv Sci (Weinh). (2024) 11(38):e2309752. doi: 10.1002/advs.202309752. PMID: 39119903 PMC11481374

[11] Ulland TK Colonna M . TREM2 - a key player in microglial biology and Alzheimer disease. Nat Rev Neurol. (2018) 14(11):667–75. doi: 10.1038/s41582-018-0072-1. PMID: 30266932

[12] Ulland TK Song WM Huang SC Ulrich JD Sergushichev A Beatty WL . TREM2 maintains microglial metabolic fitness in Alzheimer's disease. Cell. (2017) 170(4):649–663.e13. doi: 10.1016/j.cell.2017.07.023. PMID: 28802038 PMC5573224

[13] Yin S Chi X Wan F Li Y Zhou Q Kou L . TREM2 signaling in Parkinson's disease: regulation of microglial function and α-synuclein pathology. Int Immunopharmacol. (2024) 143(Pt 2):113446. doi: 10.1016/j.intimp.2024.113446. PMID: 39490141

[14] Wang Y Cella M Mallinson K Ulrich JD Young KL Robinette ML . TREM2 lipid sensing sustains the microglial response in an Alzheimer's disease model. Cell. (2015) 160(6):1061–71. doi: 10.1016/j.cell.2015.01.049. PMID: 25728668 PMC4477963

[15] Bian JH Yuan CZ Gu JX Lin WF Xiong JQ Tang ZW . TREM2 modulates macrophage pyroptosis and inflammatory responses to ameliorate aortic valve calcification. Int Immunopharmacol. (2025) 149:114161. doi: 10.1016/j.intimp.2025.114161. PMID: 39908805

[16] Li D Pan L Chen M Zhang X Jiang Z . TREM2 protects against LPS-induced murine acute lung injury through suppressing macrophage ferroptosis. Int Immunopharmacol. (2025) 150:114247. doi: 10.1016/j.intimp.2025.114247. PMID: 39946766

[17] Luo Q Deng D Li Y Shi H Zhao J Qian Q . TREM2 insufficiency protects against pulmonary fibrosis by inhibiting M2 macrophage polarization. Int Immunopharmacol. (2023) 118:110070. doi: 10.1016/j.intimp.2023.110070. PMID: 37003186

[18] Zhang Y Liu Y Luo S Liang H Guo C Du Y . An adoptive cell therapy with TREM2-overexpressing macrophages mitigates the transition from acute kidney injury to chronic kidney disease. Clin Transl Med. (2025) 15(3):e70252. doi: 10.1002/ctm2.70252. PMID: 40000418 PMC11859120

[19] Subramanian A Vernon KA Zhou Y Marshall JL Alimova M Arevalo C . Protective role for kidney TREM2(high) macrophages in obesity- and diabetes-induced kidney injury. Cell Rep. (2024) 43(6):114253. doi: 10.1016/j.celrep.2024.114253. PMID: 38781074 PMC11249042

[20] Wiernicki B Dubois H Tyurina YY Hassannia B Bayir H Kagan VE . Excessive phospholipid peroxidation distinguishes ferroptosis from other cell death modes including pyroptosis. Cell Death Dis. (2020) 11(10):922. doi: 10.1038/s41419-020-03118-0. PMID: 33110056 PMC7591475

[21] Maremonti F Meyer C Linkermann A . Mechanisms and models of kidney tubular necrosis and nephron loss. J Am Soc Nephrol. (2022) 33(3):472–86. doi: 10.1681/asn.2021101293. PMID: 35022311 PMC8975069

[22] Kilkenny C Browne WJ Cuthill IC Emerson M Altman DG . Improving bioscience research reporting: the ARRIVE guidelines for reporting animal research. PloS Biol. (2010) 8(6):e1000412. doi: 10.1371/journal.pbio.1000412. PMID: 20613859 PMC2893951

[23] Yang T Zhang S Yuan H Wang Y Cai L Chen H . Platinum-based TREM2 inhibitor suppresses tumors by remodeling the immunosuppressive microenvironment. Angew Chem Int Ed Engl. (2023) 62(2):e202213337. doi: 10.1002/anie.202213337. PMID: 36259513

[24] Chen S Zhou Y Chen Y Gu J . fastp: an ultra-fast all-in-one FASTQ preprocessor. Bioinformatics. (2018) 34(17):i884–90. doi: 10.1093/bioinformatics/bty560. PMID: 30423086 PMC6129281

[25] Kim D Langmead B Salzberg SL . HISAT: a fast spliced aligner with low memory requirements. Nat Methods. (2015) 12(4):357–60. doi: 10.1038/nmeth.3317. PMID: 25751142 PMC4655817

[26] Melo Ferreira R Sabo AR Winfree S Collins KS Janosevic D Gulbronson CJ . Integration of spatial and single-cell transcriptomics localizes epithelial cell-immune cross-talk in kidney injury. JCI Insight. (2021) 6(12):e147703. doi: 10.1172/jci.insight.147703. PMID: 34003797 PMC8262485

[27] Wang W Zhang M Ren X Song Y Xu Y Zhuang K . Single-cell dissection of cellular and molecular features underlying mesenchymal stem cell therapy in ischemic acute kidney injury. Mol Ther. (2023) 31(10):3067–83. doi: 10.1016/j.ymthe.2023.07.024. PMID: 37533253 PMC10556187

[28] Rudman-Melnick V Adam M Potter A Chokshi SM Ma Q Drake KA . Single-cell profiling of AKI in a murine model reveals novel transcriptional signatures, profibrotic phenotype, and epithelial-to-stromal crosstalk. J Am Soc Nephrol. (2020) 31(12):2793–814. doi: 10.1681/asn.2020010052. PMID: 33115917 PMC7790221

[29] Balzer MS Doke T Yang YW Aldridge DL Hu H Mai H . Single-cell analysis highlights differences in druggable pathways underlying adaptive or fibrotic kidney regeneration. Nat Commun. (2022) 13(1):4018. doi: 10.1038/s41467-022-31772-9. PMID: 35821371 PMC9276703

[30] Liao J Yu Z Chen Y Bao M Zou C Zhang H . Single-cell RNA sequencing of human kidney. Sci Data. (2020) 7(1):4. doi: 10.1038/s41597-019-0351-8. PMID: 31896769 PMC6940381

[31] Lake BB Menon R Winfree S Hu Q Melo Ferreira R Kalhor K . An atlas of healthy and injured cell states and niches in the human kidney. Nature. (2023) 619(7970):585–94. doi: 10.1038/s41586-023-05769-3. PMID: 37468583 PMC10356613

[32] Kemp SB Steele NG Carpenter ES Donahue KL Bushnell GG Morris AH . Pancreatic cancer is marked by complement-high blood monocytes and tumor-associated macrophages. Life Sci Alliance. (2021) 4(6):e202000935. doi: 10.26508/lsa.202000935. PMID: 33782087 PMC8091600

[33] Ikeda N Asano K Kikuchi K Uchida Y Ikegami H Takagi R . Emergence of immunoregulatory Ym1+Ly6Chi monocytes during recovery phase of tissue injury. Sci Immunol. (2018) 3(28):eaat0207. doi: 10.1126/sciimmunol.aat0207. PMID: 30291130

[34] Jin G Guo N Liu Y Zhang L Chen L Dong T . 5-aminolevulinate and CHIL3/CHI3L1 treatment amid ischemia aids liver metabolism and reduces ischemia-reperfusion injury. Theranostics. (2023) 13(14):4802–20. doi: 10.7150/thno.83163. PMID: 37771779 PMC10526663

[35] To SKY Tang MKS Tong Y Zhang J Chan KKL Ip PPC . A selective β-catenin-metadherin/CEACAM1-CCL3 axis mediates metastatic heterogeneity upon tumor-macrophage interaction. Advanced Sci (Weinheim Baden-Wurttemberg Germany). (2022) 9(16):e2103230. doi: 10.1002/advs.202103230. PMID: 35403834 PMC9165500

[36] Fu Y Pajulas A Wang J Zhou B Cannon A Cheung CCL . Mouse pulmonary interstitial macrophages mediate the pro-tumorigenic effects of IL-9. Nat Commun. (2022) 13(1):3811. doi: 10.1038/s41467-022-31596-7. PMID: 35778404 PMC9249769

[37] Juric V Mayes E Binnewies M Lee T Canaday P Pollack JL . TREM1 activation of myeloid cells promotes antitumor immunity. Sci Transl Med. (2023) 15(711):eadd9990. doi: 10.1126/scitranslmed.add9990. PMID: 37647386

[38] Yurdagul A Subramanian M Wang X Crown SB Ilkayeva OR Darville L . Macrophage metabolism of apoptotic cell-derived arginine promotes continual efferocytosis and resolution of injury. Cell Metab. (2020) 31(3):518. doi: 10.1016/j.cmet.2020.01.001. PMID: 32004476 PMC7173557

[39] Livingston MJ Shu S Fan Y Li Z Jiao Q Yin XM . Tubular cells produce FGF2 via autophagy after acute kidney injury leading to fibroblast activation and renal fibrosis. Autophagy. (2023) 19(1):256–77. doi: 10.1080/15548627.2022.2072054. PMID: 35491858 PMC9809951

[40] Livingston MJ Zhang M Kwon SH Chen JK Li H Manicassamy S . Autophagy activates EGR1 via MAPK/ERK to induce FGF2 in renal tubular cells for fibroblast activation and fibrosis during maladaptive kidney repair. Autophagy. (2024) 20(5):1032–53. doi: 10.1080/15548627.2023.2281156. PMID: 37978868 PMC11135847

[41] Shao X Gomez CD Kapoor N Considine JM Grams C Gao YT . MatrisomeDB 2.0: 2023 updates to the ECM-protein knowledge database. Nucleic Acids Res. (2023) 51(D1):D1519–30. doi: 10.1093/nar/gkac1009. PMID: 36399478 PMC9825471

[42] Shao X Taha IN Clauser KR Gao YT Naba A . MatrisomeDB: the ECM-protein knowledge database. Nucleic Acids Res. (2020) 48(D1):D1136–44. doi: 10.1093/nar/gkz849. PMID: 31586405 PMC6943062

[43] Toyonaga K Torigoe S Motomura Y Kamichi T Hayashi JM Morita YS . C-type lectin receptor DCAR recognizes mycobacterial phosphatidyl-inositol mannosides to promote a Th1 response during infection. Immunity. (2016) 45(6):1245–57. doi: 10.1016/j.immuni.2016.10.012. PMID: 27887882

[44] Li Z Li L Yue M Peng Q Pu X Zhou Y . Tracing immunological interaction in trimethylamine N-oxide hydrogel-derived zwitterionic microenvironment during promoted diabetic wound regeneration. Advanced Materials (Deerfield Beach Fla). (2024) 36(33):e2402738. doi: 10.1002/adma.202402738. PMID: 38885961

[45] Pyonteck SM Akkari L Schuhmacher AJ Bowman RL Sevenich L Quail DF . CSF-1R inhibition alters macrophage polarization and blocks glioma progression. Nat Med. (2013) 19(10):1264–72. doi: 10.1038/nm.3337. PMID: 24056773 PMC3840724

[46] Ries CH Cannarile MA Hoves S Benz J Wartha K Runza V . Targeting tumor-associated macrophages with anti-CSF-1R antibody reveals a strategy for cancer therapy. Cancer Cell. (2014) 25(6):846–59. doi: 10.1016/j.ccr.2014.05.016. PMID: 24898549

[47] Gao S Zhou J Liu N Wang L Gao Q Wu Y . Curcumin induces M2 macrophage polarization by secretion IL-4 and/or IL-13. J Mol Cell Cardiol. (2015) 85:131–9. doi: 10.1016/j.yjmcc.2015.04.025. PMID: 25944087

[48] Schibalski RS Shulha AS Tsao BP Palygin O Ilatovskaya DV . The role of polyamine metabolism in cellular function and physiology. Am J Physiol Cell Physiol. (2024) 327(2):C341–56. doi: 10.1152/ajpcell.00074.2024. PMID: 38881422 PMC11427016

[49] Ouyang Q Wang C Sang T Tong Y Zhang J Chen Y . Depleting profibrotic macrophages using bioactivated *in vivo* assembly peptides ameliorates kidney fibrosis. Cell Mol Immunol. (2024) 21(8):826–41. doi: 10.1038/s41423-024-01190-6. PMID: 38871810 PMC11291639

[50] Colonna M . The biology of TREM receptors. Nat Rev Immunol. (2023) 23(9):580–94. doi: 10.1038/s41577-023-00837-1. PMID: 36750615 PMC9904274

[51] Jaitin DA Adlung L Thaiss CA Weiner A Li B Descamps H . Lipid-associated macrophages control metabolic homeostasis in a Trem2-dependent manner. Cell. (2019) 178(3):686–698.e14. doi: 10.1016/j.cell.2019.05.054. PMID: 31257031 PMC7068689

[52] Deczkowska A Weiner A Amit I . The physiology, pathology, and potential therapeutic applications of the TREM2 signaling pathway. Cell. (2020) 181(6):1207–17. doi: 10.1016/j.cell.2020.05.003. PMID: 32531244

[53] Che Y Yu Z Ji S Yang D . Decoding TREM2 signaling pathways: Linking macrophage glycolysis to inflammatory diseases in the CNS. Neurol Neuroimmunol Neuroinflamm. (2026) 13(2):e200527. doi: 10.1212/nxi.0000000000200527. PMID: 41512220 PMC12794546

[54] Zhang J St Pierre Schneider B Muguerza E Chung E Hsu CG . Synergistic potential of TREM2 agonists and exercise training in Alzheimer's disease. Am J Physiol Endocrinol Metab. (2026) 330(3):E298–e308. doi: 10.1152/ajpendo.00124.2025. PMID: 41494649

[55] DeBose-Boyd RA Ye J . SREBPs in lipid metabolism, insulin signaling, and beyond. Trends Biochem Sci. (2018) 43(5):358–68. doi: 10.1016/j.tibs.2018.01.005. PMID: 29500098 PMC5923433

[56] Zheng H Jia L Liu CC Rong Z Zhong L Yang L . TREM2 promotes microglial survival by activating Wnt/β-catenin pathway. J Neurosci. (2017) 37(7):1772–84. doi: 10.1523/jneurosci.2459-16.2017. PMID: 28077724 PMC5320608

[57] Zhang T Pang W Feng T Guo J Wu K Nunez Santos M . TMEM106B regulates microglial proliferation and survival in response to demyelination. Sci Adv. (2023) 9(18):eadd2676. doi: 10.1126/sciadv.add2676. PMID: 37146150 PMC10162677

[58] Cheng B Li X Dai K Duan S Rong Z Chen Y . Triggering receptor expressed on myeloid cells-2 (TREM2) interacts with colony-stimulating factor 1 receptor (CSF1R) but is not necessary for CSF1/CSF1R-mediated microglial survival. Front Immunol. (2021) 12:633796. doi: 10.3389/fimmu.2021.633796. PMID: 33841415 PMC8027073

[59] Liu W Taso O Wang R Bayram S Graham AC Garcia-Reitboeck P . Trem2 promotes anti-inflammatory responses in microglia and is suppressed under pro-inflammatory conditions. Hum Mol Genet. (2020) 29(19):3224–48. doi: 10.1093/hmg/ddaa209. PMID: 32959884 PMC7689298

[60] Shi Y Holtzman DM . Interplay between innate immunity and Alzheimer disease: APOE and TREM2 in the spotlight. Nat Rev Immunol. (2018) 18(12):759–72. doi: 10.1038/s41577-018-0051-1. PMID: 30140051 PMC6425488

[61] Zou W Reeve JL Liu Y Teitelbaum SL Ross FP . DAP12 couples c-Fms activation to the osteoclast cytoskeleton by recruitment of Syk. Mol Cell. (2008) 31(3):422–31. doi: 10.1016/j.molcel.2008.06.023. PMID: 18691974 PMC2584874

[62] Otero K Turnbull IR Poliani PL Vermi W Cerutti E Aoshi T . Macrophage colony-stimulating factor induces the proliferation and survival of macrophages via a pathway involving DAP12 and beta-catenin. Nat Immunol. (2009) 10(7):734–43. doi: 10.1038/ni.1744. PMID: 19503107 PMC4004764

[63] Cui Y Chen C Tang Z Yuan W Yue K Cui P . TREM2 deficiency aggravates renal injury by promoting macrophage apoptosis and polarization via the JAK-STAT pathway in mice. Cell Death Dis. (2024) 15(6):401. doi: 10.1038/s41419-024-06756-w. PMID: 38849370 PMC11161629

[64] Wu K Byers DE Jin X Agapov E Alexander-Brett J Patel AC . TREM-2 promotes macrophage survival and lung disease after respiratory viral infection. J Exp Med. (2015) 212(5):681–97. doi: 10.1084/jem.20141732. PMID: 25897174 PMC4419356

[65] Zhong L Chen XF Wang T Wang Z Liao C Wang Z . Soluble TREM2 induces inflammatory responses and enhances microglial survival. J Exp Med. (2017) 214(3):597–607. doi: 10.1084/jem.20160844. PMID: 28209725 PMC5339672

[66] Jung SH Hwang BH Shin S Park EH Park SH Kim CW . Spatiotemporal dynamics of macrophage heterogeneity and a potential function of Trem2(hi) macrophages in infarcted hearts. Nat Commun. (2022) 13(1):4580. doi: 10.1038/s41467-022-32284-2. PMID: 35933399 PMC9357004

[67] Long H Simmons A Mayorga A Burgess B Nguyen T Budda B . Preclinical and first-in-human evaluation of AL002, a novel TREM2 agonistic antibody for Alzheimer's disease. Alzheimers Res Ther. (2024) 16(1):235. doi: 10.1186/s13195-024-01599-1. PMID: 39444037 PMC11515656

[68] van Lengerich B Zhan L Xia D Chan D Joy D Park JI . A TREM2-activating antibody with a blood-brain barrier transport vehicle enhances microglial metabolism in Alzheimer's disease models. Nat Neurosci. (2023) 26(3):416–29. doi: 10.1038/s41593-022-01240-0. PMID: 36635496 PMC9991924

