# Supplementary material for: Trem2 activation by renal tubular debris sustains Arg1+ macrophage survival and promotes tubular epithelial repair in renal ischemia–reperfusion injury
Source: Front Immunol. 2026 Apr 10;17:1819941. doi: 10.3389/fimmu.2026.1819941 (PMC13106072; doi:10.3389/fimmu.2026.1819941)
Supplement: Supplementary Figure 1 — Temporal dynamics and subpopulations of monocyte/macrophages during IRI. [file DataSheet1.pdf]

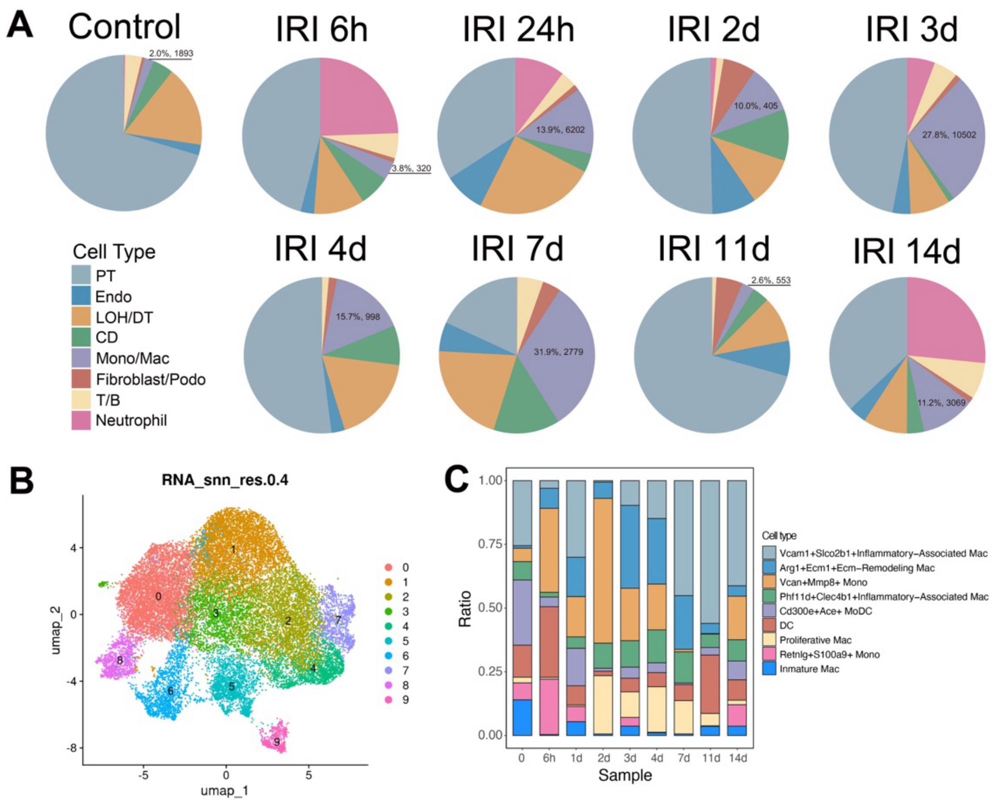

Supplementary Figure S1. Temporal dynamics and subpopulations of monocyte/macrophages during IRI.

(A) Temporal dynamics of the monocyte/macrophage cell population during IRI. The proportion of Mono/Mac cells increased from 2.0% in the control group to 13.9% at 24 hours post-IRI, and reached 27.8% and 31.9% at 3 and 7 days post-IRI, respectively. **(B)** UMAP-based clustering analysis of 25,343 monocyte/macrophage cells, revealing 10 distinct clusters. **(C)** Proportion analysis of monocyte/macrophage subpopulations at different time points post-IRI revealed that the *Arg1*^{hi}*Ecm1*^{hi} ECM-Remodeling Mac subpopulation peaked at day 3 post-IRI.
