# Supplementary material for: Trem2 activation by renal tubular debris sustains Arg1+ macrophage survival and promotes tubular epithelial repair in renal ischemia–reperfusion injury
Source: Front Immunol. 2026 Apr 10;17:1819941. doi: 10.3389/fimmu.2026.1819941 (PMC13106072; doi:10.3389/fimmu.2026.1819941)
Supplement: Supplementary Figure 2 — Co-expression patterns of Arg1, Trem2, Spp1, and Apoe at different time points following IRI. [file DataSheet2.pdf]

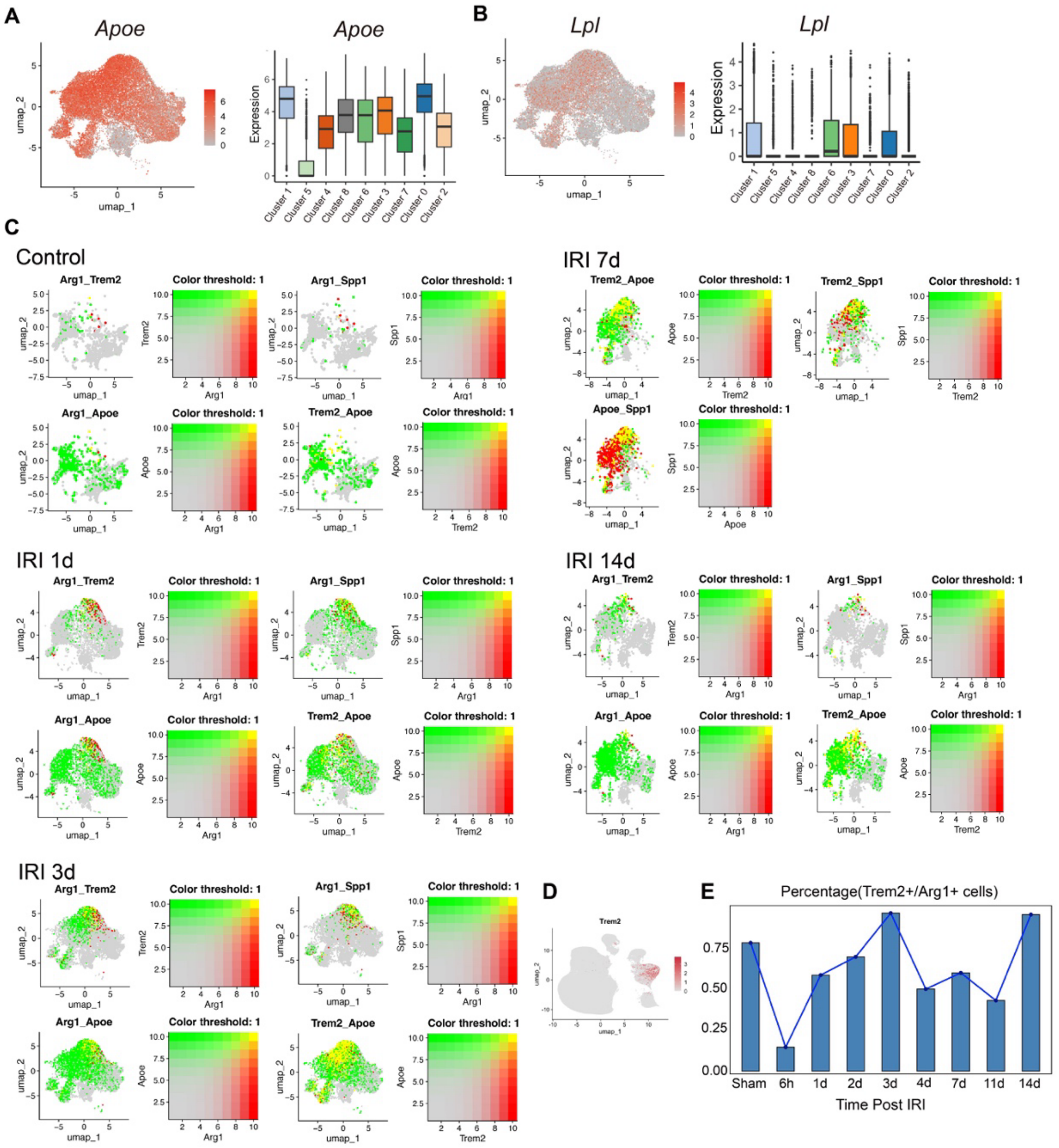

Supplementary Figure S2. Co-expression patterns of *Arg1*, *Trem2*, *Spp1*, and *Apoe* at different time points following IRI.

(A, B) The FeaturePlot demonstrates the expression patterns of *Apoe* and *Lpl* across monocyte/macrophage subpopulations, highlighting their lack of specificity.**(C)** Co-expression of Arg1 with Trem2, Apoe, and Spp1, as well as co-expression of Trem2 with Apoe, was barely detectable in the control group, but significantly increased on days 1, 3, 7, and 14 post-IRI. **(D)** The FeaturePlot shows that Trem2 is specifically expressed in the monocyte/macrophage subset among all cell populations post-IRI. **(E)** The percentage of Trem2-positive expression in the $Arg1^{hi}Ecm1^{hi}$ ECM-Remodeling Mac subpopulation peaks on day 3 post-IRI.
