# Supplementary material for: Trem2 activation by renal tubular debris sustains Arg1+ macrophage survival and promotes tubular epithelial repair in renal ischemia–reperfusion injury
Source: Front Immunol. 2026 Apr 10;17:1819941. doi: 10.3389/fimmu.2026.1819941 (PMC13106072; doi:10.3389/fimmu.2026.1819941)
Supplement: Supplementary Figure 3 — Activation of the Apoe-Trem2 signaling axis in Arg1hiEcm1hiECM-Remodeling Macrophages after IRI. [file DataSheet3.pdf]

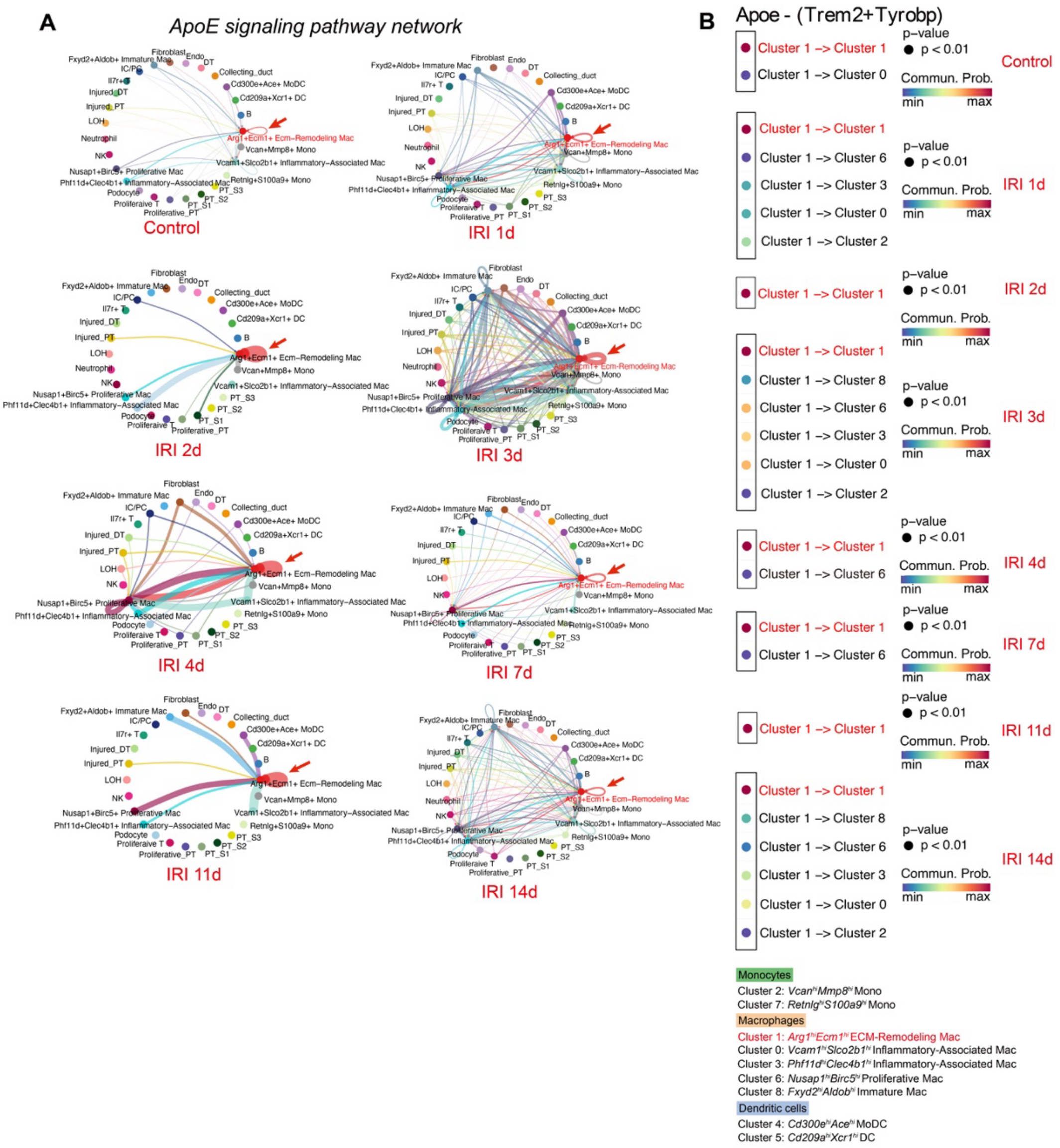


**Supplementary Figure S3. Activation of the Apoe-Trem2 signaling axis in *Arg1^hi^Ecm1^hi^* ECM-Remodeling Macrophages after IRI.**

**(A)** Apoe signaling was significantly activated in the *Arg1*$^{hi}$*Ecm1*$^{hi}$ ECM-Remodeling Mac at days 1, 2, 3, 4, 7, 11 and 14 post-injury, as compared to the control group, which showed no significant activation in the same macrophage subset. **(B)** Apoe signaling primarily involved Apoe-Trem2/Tyrobp interactions, which were most pronounced in *Arg1*$^{hi}$*Ecm1*$^{hi}$ ECM-Remodeling Mac (Cluster 1).

.
