# Supplementary material for: Trem2 activation by renal tubular debris sustains Arg1+ macrophage survival and promotes tubular epithelial repair in renal ischemia–reperfusion injury
Source: Front Immunol. 2026 Apr 10;17:1819941. doi: 10.3389/fimmu.2026.1819941 (PMC13106072; doi:10.3389/fimmu.2026.1819941)
Supplement: Supplementary Figure 4 — Trem2 is upregulated early after IRI and co-localizes with Arg1 in macrophages. [file DataSheet4.pdf]

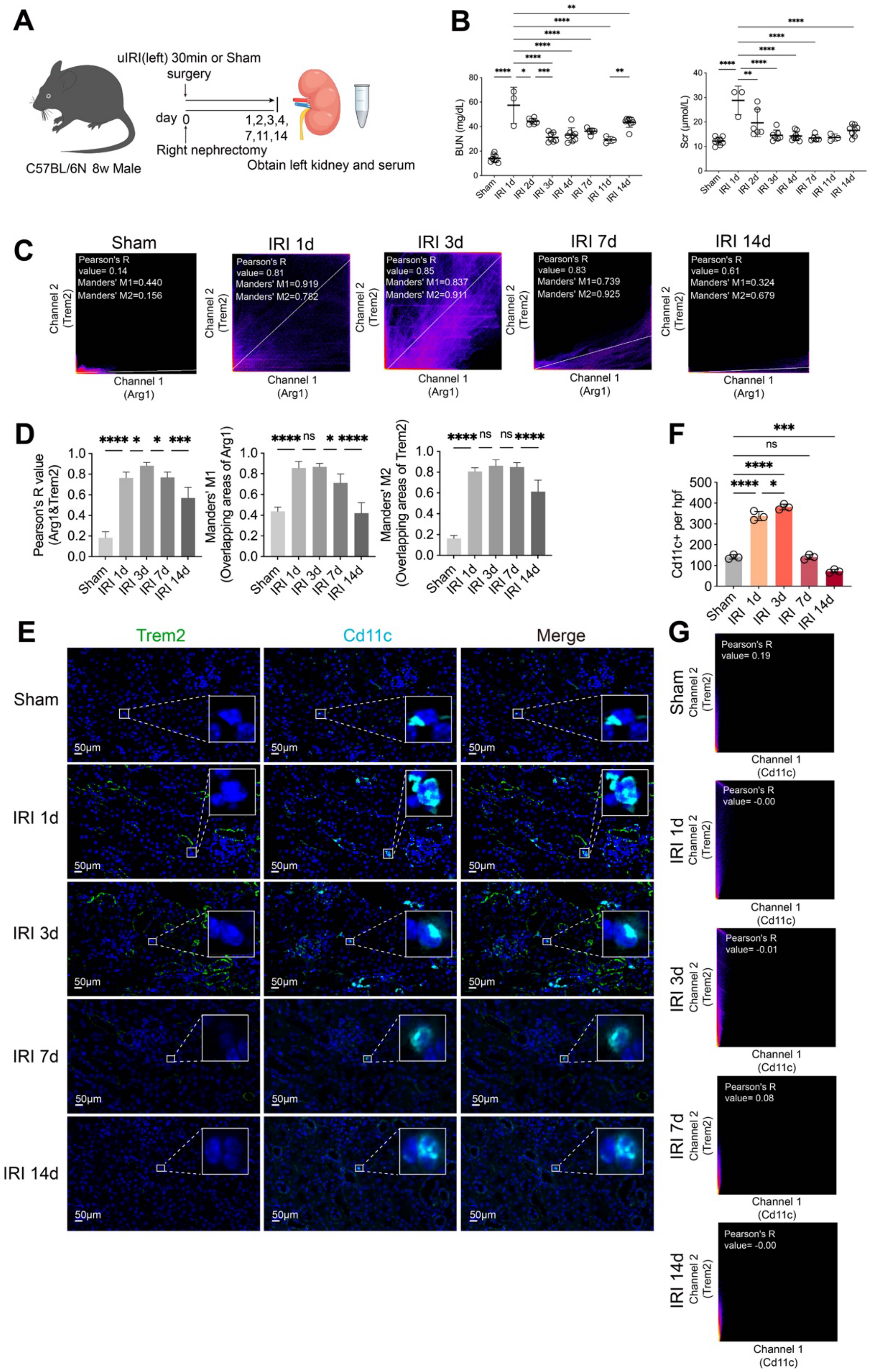

Supplementary Figure S4. Trem2 is upregulated early after IRI and co-localizes with Arg1 in macrophages.

(B) Serum BUN and Scr rose sharply at day 1 post-IRI, partially recovered by day 2, and improved further at day 3, indicating activation of repair. Levels normalized thereafter but increased again at day 14, suggesting transition toward CKD. **(C)** HE staining revealed pronounced tubular injury at days 1 and 3 post-IRI, whereas Masson's trichrome and Sirius Red staining demonstrated the onset of interstitial fibrosis from day 7. **(C, D)** Co-localization analysis confirmed significant overlap of Trem2 and Arg1 expression at days 1, 3, and 7. **(E, F, G)** Double IF staining demonstrates that the infiltration of $Cd11c^+$ cells peaks at day 3 post-IRI and gradually declines thereafter, and that Trem2 and Cd11c exhibit no co-expression in IRI kidneys, verifying the exclusive expression of Trem2 in macrophages instead of the dendritic cell population. Significance was evaluated using Student's unpaired *t* test and one-way ANOVA followed by Tukey's test. $^{*}P < 0.05$, $^{**}P < 0.01$, $^{***}P < 0.001$, $^{****}P < 0.0001$, *ns*: no significance.
