# Supplementary material for: Trem2 activation by renal tubular debris sustains Arg1+ macrophage survival and promotes tubular epithelial repair in renal ischemia–reperfusion injury
Source: Front Immunol. 2026 Apr 10;17:1819941. doi: 10.3389/fimmu.2026.1819941 (PMC13106072; doi:10.3389/fimmu.2026.1819941)
Supplement: Supplementary Figure 5 — Representative FACS gating strategies. [file DataSheet5.pdf]

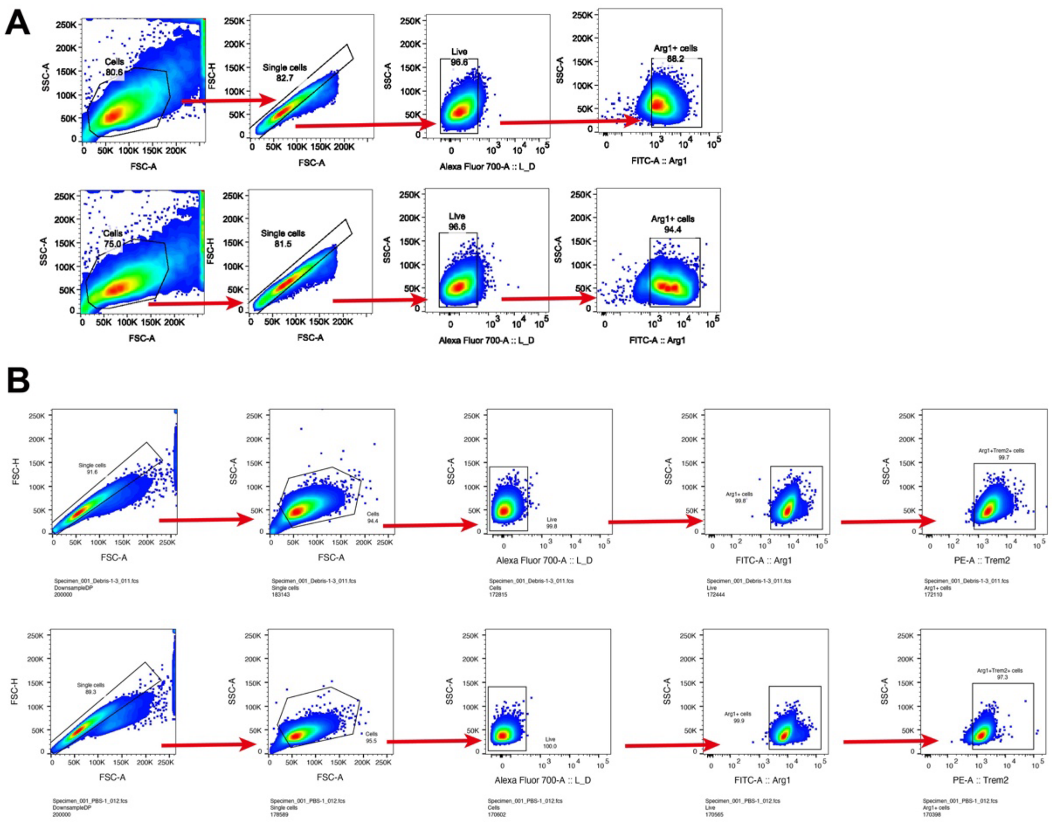

Supplementary Figure S5. Representative FACS gating strategies.

(A) Representative gating strategy showing the sequential exclusion of debris, doublets, and dead cells to identify the Arg1^{+} population. **(B)** Representative gating strategy illustrating the hierarchical identification of the Trem2^{+} Arg1^{+} subpopulation following the exclusion of debris, doublets, and dead cells.
