# Supplementary material for: Trem2 activation by renal tubular debris sustains Arg1+ macrophage survival and promotes tubular epithelial repair in renal ischemia–reperfusion injury
Source: Front Immunol. 2026 Apr 10;17:1819941. doi: 10.3389/fimmu.2026.1819941 (PMC13106072; doi:10.3389/fimmu.2026.1819941)
Supplement: Supplementary Figure 6 — Original Western blot images (Trem2, Arg1). [file DataSheet6.pdf]

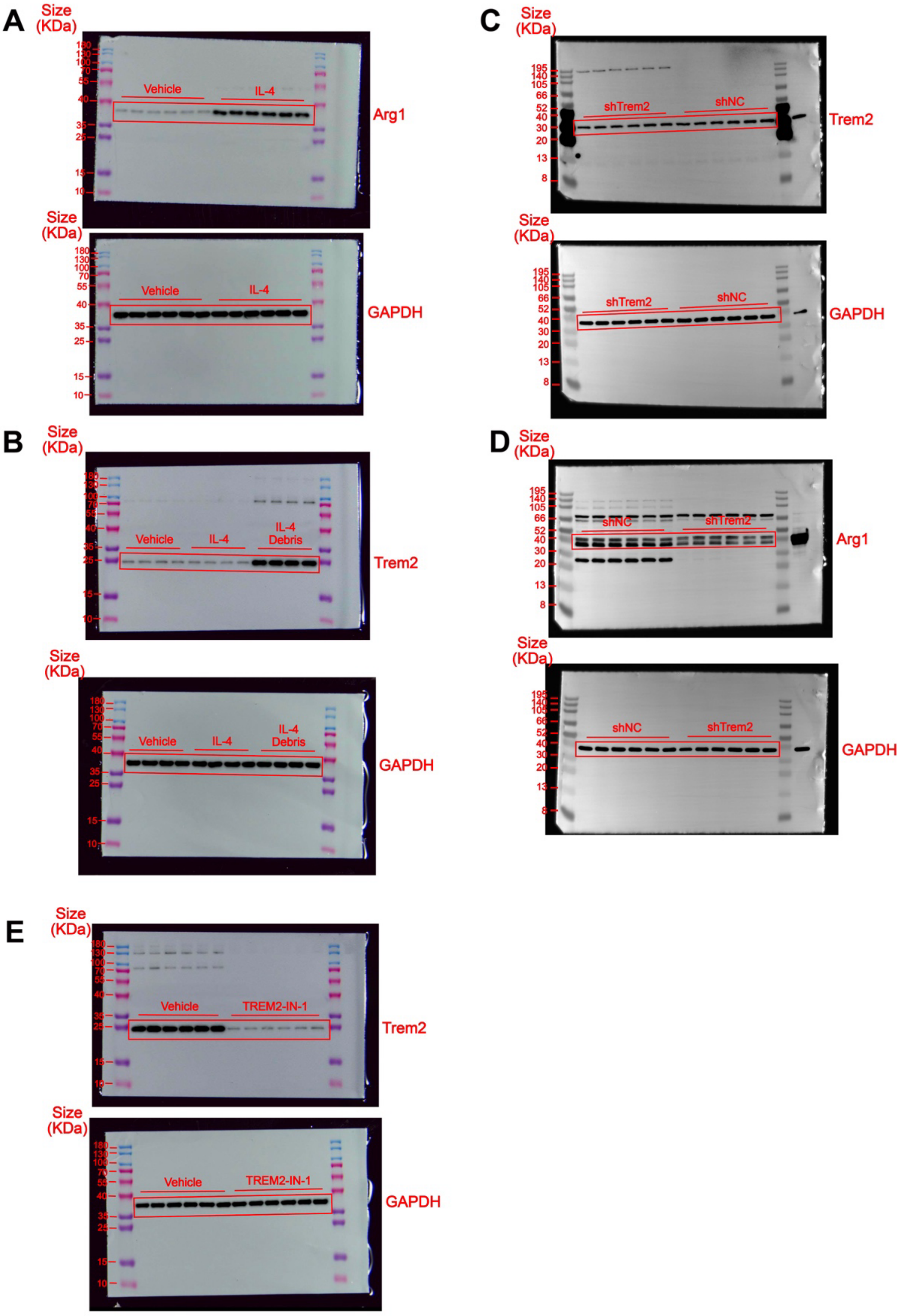

Supplementary Figure S6. Original Western blot images (Trem2, Arg1).

(A) Western blot analysis showed that IL-4 stimulation significantly upregulated Arg1 expression in BMDMs. **(B)** Western blot analysis showed that treatment with renal tubular debris induced upregulated Trem2 expression in $Arg1^{high}$ BMDMs.

(C) Western blot analysis confirmed the stable knockdown of Trem2 protein in RAW264.7 cells. **(D)** Western blot analysis confirmed that Trem2 knockdown macrophages exhibited markedly reduced Arg1 expression following IL-4 treatment. **(E)** Western blot analysis showed that treatment with TREM2-IN-1 markedly reduced Trem2 protein expression in BMDMs.
