# Supplementary material for: Trem2 activation by renal tubular debris sustains Arg1+ macrophage survival and promotes tubular epithelial repair in renal ischemia–reperfusion injury
Source: Front Immunol. 2026 Apr 10;17:1819941. doi: 10.3389/fimmu.2026.1819941 (PMC13106072; doi:10.3389/fimmu.2026.1819941)
Supplement: Supplementary Figure 7 — Original Western blot images (Pten, Bcl2). [file DataSheet7.pdf]

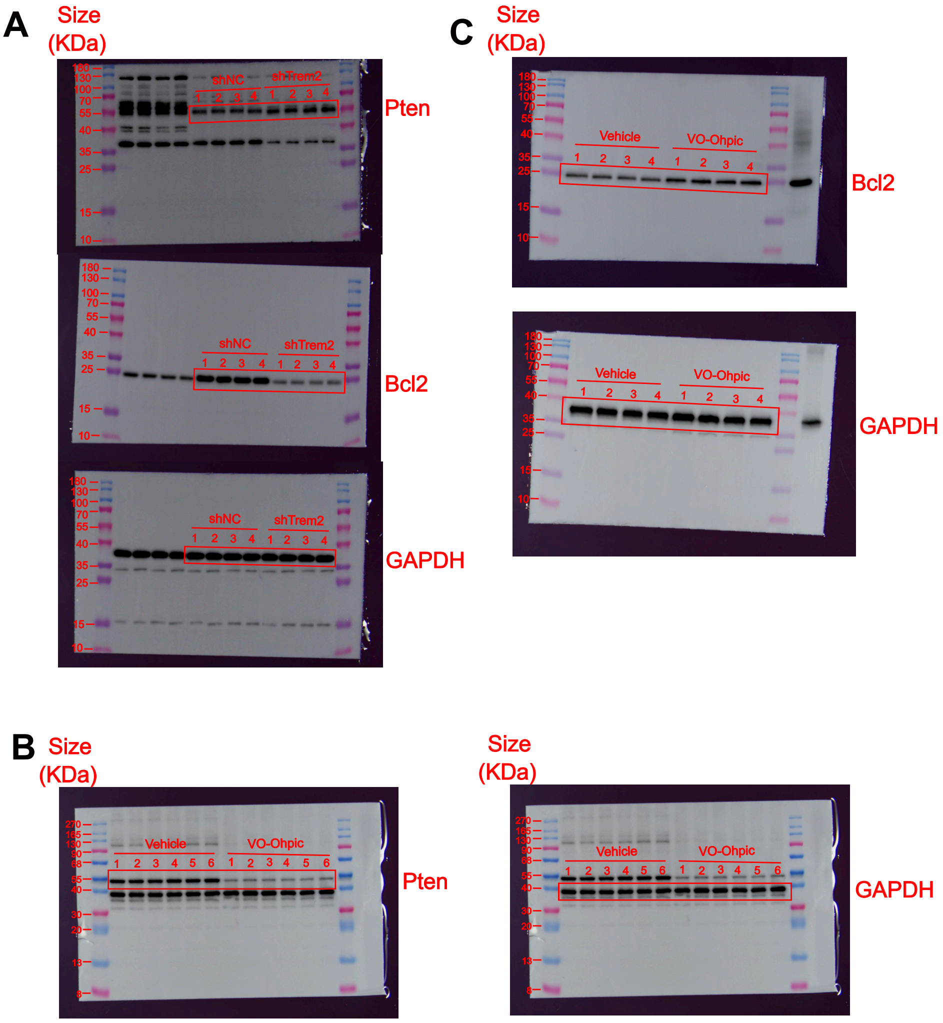

Supplementary Figure S7. Original Western blot images (Pten, Bcl2).

(A) Original Western blot images demonstrate elevated Pten and reduced Bcl2 expression in Trem2-knockdown Arg1^{+} macrophages. **(B)** Original Western blot images showed that VO-Ohpic treatment markedly suppressed Pten expression in Trem2-knockdown Arg1^{+} macrophages. **(C)** Original Western blot images showed that, under co-culture conditions, Bcl2 protein expression was restored in VO-Ohpic-treated cells.
