# Supplementary material for: Trem2 activation by renal tubular debris sustains Arg1+ macrophage survival and promotes tubular epithelial repair in renal ischemia–reperfusion injury
Source: Front Immunol. 2026 Apr 10;17:1819941. doi: 10.3389/fimmu.2026.1819941 (PMC13106072; doi:10.3389/fimmu.2026.1819941)
Supplement: Supplementary file 8 [file DataSheet8.pdf]

Supplementary Methods

scRNA-seq data processing

The R package Seurat (v5.3.0)[1] was applied to analyze the scRNA-seq data. The above samples were integrated and used for downstream analysis. For quality control, cells with 200 ≤ gene numbers (nFeature_RNA) ≤ 6000, mitochondrial gene percentage ≤ 20% were retained. A total of 243,350 high-quality cells passed the quality control filtering and were utilized for subsequent analysis. The single cell count data matrix was normalized using the “NormalizeData” function. The top 2000 highly variable features were selected for principal component analysis (PCA) dimensionality reduction via the “RunPCA” function. For batch correction, we applied the “RunHarmony” function in the R package harmony (v1.2.3)[2] to integrate different samples and eliminate the technical batch variations across datasets. Then we used “FindNeighbors” and “FindClusters” to cluster the cells. The R package clustree (v0.5.1)[3] was applied to show the relationships between clusters and determine the clustering resolution. “RunUMAP” was used to visualize the dimension reduction plots of cells.

Cell type annotation

We identified the marker genes of each cluster by running the “FindAllMarkers” algorithm (|logFC| > 0.3, Minpct = 0.25) and annotated the clusters manually based on the expression of cell-specific genes acquired from the CellMarker2.0 database and previously reported studies[4-7].

Differential gene expression analysis

scRNA-seq: Pseudobulk transcriptional profiling was conducted using DESeq2 (v1.40.0)[8]. The gene expression matrix of monocytes and macrophages were aggregated from the Seurat object by applying the aggregateExpression function to extract raw counts from the 'RNA' assay. Gene-level counts were summed across cells within each biological sample to generate a consolidated expression matrix. Normalization was performed using DESeq2's default pipeline, including library size factor estimation via the estimateSizeFactors function and variance stabilization using the vst transformation. The resultant normalized matrix mirrored conventional bulk RNA-seq data structures. Genes with adjusted P-value < 0.05 and $|log_2FC| > 1$ were considered significant.

Bulk RNA-seq: DEGs between IRI and control groups were identified using the DEseq2 package (v1.40.0). Genes with adjusted *P*-value < 0.05 and FC > 1.5 were considered significant.

Developmental trajectory analysis

Monocle3 package(v1.3.7)[9] was used to order cells along a developmental trajectory, modeling their differentiation over time. We set monocytes and normal renal tubular epithelial cells as the root of trajectory in "order_cells" for Mono/Mac and renal tubular epithelial cells respectively based on current studies.

Cell-cell communication analysis

The R package CellChat (v2.1.2)[10] was applied to infer the ligand-receptor interactions among different cell clusters. To compare the intercellular communication networks among

different time points, we ran CellChat on gene expression matrices of different time points separately. Ligand-receptor interactions with p-value < 0.05 were considered significant.

Enrichment analysis

Gene functional annotation was performed using the clusterProfiler package (v4.14.6), including GO, KEGG, and GSEA analyses. Only biological process terms with a p-value < 0.05 were retained for GO analysis. For KEGG pathway enrichment, differentially expressed genes were mapped to the KEGG database, and significantly enriched pathways were identified using a hypergeometric test with a Benjamini-Hochberg adjusted p-value < 0.05. GSEA was conducted using the hallmark and KEGG gene sets from the MSigDB database. Results were visualized using ggplot2.

Pten inhibition in Arg1$^+$ macrophages using VO-Ohpic

Control and shTrem2 knockdown Arg1$^+$ macrophages, as well as Trem2 inhibitor TREM2-IN-1–treated Arg1^{high} BMDMs, were cultured in 24-well plates at a density of 6 × 10^4 cells per well. Cells were pretreated with 10 μM of the PTEN-specific inhibitor VO-Ohpic (MCE, HY-13074, USA) for 24 hours. Following this, 2 × 10^4 renal tubular debris were added to each well, and cells were cultured for an additional 24 hours before proceeding with subsequent analysis.

RT-qPCR analysis

All real-time reactions were performed on a QuantStudio™ 1 Plus System (Thermo Fisher Scientific, USA) using the following primers: Apoe: 5'-CCTGAACCGCTTCTGGGATT-3' (Forward) and 5'-CCATCAGTGCCGTCAGTTCT-3' (Reverse); Spp1: 5'-CTGCAGTTCTCCTGGCTGAA-3' (Forward) and 5'-TCTGGGTGCAGGCTGTA AAG-3' (Reverse); Arg1: 5'-ACATTGGCTTGCGAGACGTA-3' (Forward) and 5'-ATCACCTTGCCAATCCCCAG-3' (Reverse); Trem2: 5'-GGGTCACCTCTAGCCTACCA-3' (Forward) and 5'-CTCTGACCACAGGTGTTCCC-3' (Reverse);. GAPDH: 5-AACTTTGGCATTGTGGAAGGGCTC-3 (Forward) and 5-TGGAAGAGTGGGAGTTGCTGTTGA-3 (Reverse).

Western blot assay

Protein concentrations were measured using the BCA Protein Assay Kit (Thermo Fisher Scientific, USA, 23221). Equal protein amounts from each sample were loaded onto an SDS-polyacrylamide gel for electrophoresis. After blocking with 5% non-fat milk, the membranes were incubated overnight at 4°C with primary antibodies, followed by incubation with a fluorescently-labeled secondary antibody. The primary antibodies used were Trem2 (Bioss, China, bs-2723R, 1: 1000 dilution), Arg1 (Proteintech Group, Inc., 66129-1-Ig, 1: 1000 dilution), Pten (Servicebio, GB113803, 1: 1000 dilution), Bcl2 (Servicebio, GB154380, 1: 1000 dilution) and GAPDH (Servicebio, GB15004, 1: 5000 dilution). Notably, all target proteins and the internal control (GAPDH) were detected on the same membrane. Since the molecular weights of the target proteins and GAPDH are similar, the internal control antibody (GAPDH)

was incubated after washing the membrane. Finally, signals were captured using an Odyssey® Dual-Color Infrared Fluorescence Imaging System (LI-COR Biosciences).

Flow cytometry analysis

Cells were first stained with the Fixable Viability Stain 700 (BD Horizon™, USA, 564997) and Purified Rat Anti-Mouse CD16/CD32 antibody (Mouse BD Fc Block) (BD Pharmingen™, USA, 553141) to remove dead cells and prevent non-specific binding. Afterward, surface staining was performed by incubating the cells with the Trem2 antibody (BioLegend, USA, 824805) for 45 minutes at 4°C. For intracellular staining, the cells were permeabilized and subsequently incubated with the Arg1 antibody (Bioss, China, bs-8585R-BF488) for 45 minutes at 4°C to detect intracellular Arg1 expression. Following incubation, the cells were washed three times with PBS. Flow cytometry was performed using the BD LSRFortessa™ (Becton, Dickinson and Company, USA), and the data were analyzed with FlowJo V10 software.

CCK-8 assay

The Cell Counting Kit-8 (CCK-8) assay was employed to evaluate cell viability. Cells were seeded at $6x10^4$ cells/well in 700 μL of medium in a 24-well plate, with three replicate wells for each group.70 μL of CCK-8 solution(Lablead, China, CK001) was added to each well, and the plate was incubated for 3 hours in a CO_2 incubator. Absorbance at 450 nm was measured using a microplate reader (Varioskan Flash 3001, Thermo Fisher Scientific).

Annexin V-PE/7AAD Staining

Cell apoptosis was assessed using the Annexin V-PE/7-AAD double staining (Solarbio, CA1030) method with flow cytometry. Cells were seeded and treated as per the experimental design. After treatment, cells were collected, washed with PBS, and resuspended to 1×10^6 cells/mL. The cells were stained with 5 μL of Annexin V-PE and 10 μL of 7-AAD solution in 100 μL of 1× Annexin V binding buffer for 15 minutes at room temperature in the dark. After staining, 400 μL of 1× Annexin V binding buffer was added, and the cells were analyzed by flow cytometry (BD LSRFortessa™). Early apoptotic cells were identified as Annexin V+/PI-, late apoptotic or necrotic cells as Annexin V+/7AAD+, necrotic cells as Annexin V-/7AAD+, and viable cells as Annexin V-/7AAD-. Flow cytometry data were analyzed using FlowJo V10 software to calculate the percentage of cells in each apoptosis stage.

ELISA assay

Culture supernatants from IL-4–stimulated RAW264.7 cells or BMDMs were collected for quantification of $Arg1^+$ macrophage–derived factors by ELISA. Apoe and Spp1 were measured using the Apoe ELISA Kit (BIOESN, China, BES1377K) and the Spp1 ELISA Kit (Multi Sciences, China, EK2135/2-AW1), respectively. Spermine levels were measured using a commercial Spermine ELISA kit (MyBioSource, China, MBS018928), while spermidine levels were determined using a commercial Spermidine ELISA kit (BAIYI Biology, China, BY-M03736). HGF was measured with the HGF ELISA Kit (BIOESN, China, BES0334K), VEGF with the VEGF ELISA Kit (Beyotime, China, PV957), and IL-10 with the IL-10 ELISA Kit (Beyotime, China, PI523), according to the manufacturers' protocols. Absorbance at 450 nm

was recorded on a multifunctional microplate reader (Varioskan Flash 3001, Thermo Fisher Scientific, USA), and concentrations were calculated from standard curves.

Histologic analysis

Kidney tissues were preserved in 4% paraformaldehyde, embedded in paraffin, and sectioned for H&E staining. Tubular injury was assessed based on the H&E staining as described in a previous study [11]. Two blinded investigators independently evaluated the extent of tubular injury (including tubular necrosis, cast formation, and loss of brush borders). Three random high-power fields were selected, and the percentage of affected tubules was graded using the following scale: 0, no injury; 1, 1%-25%; 2, 26%-50%; 3, 51%-75%; and 4, 75%-100%. In addition, Masson’s trichrome and Picrosirius Red staining were performed to assess renal fibrosis. The tissue sections were deparaffinized and rehydrated before being stained. For Picrosirius Red staining, collagen fibers were visualized under polarized light as bright red or orange. For Masson’s trichrome, the tissue was stained with iron hematoxylin for nuclei, acid fuchsin for cytoplasm, and aniline blue for collagen fibers. Collagen fibers were observed as blue, indicating fibrosis in the basement membrane and other fibrotic areas.

Multi-immunofluorescence

Fluorescent immunohistochemistry with tyramide signal amplification was conducted on 4 μm TMA sections. Slides were deparaffinized using xylene, ethanol, and 10% neutral buffered formalin, then washed in distilled water and TBST. Antigen retrieval was performed by microwave heating in citrate buffer (full power 45 seconds, 20% power 15 min). After blocking

with protein blocker (Dako, X0909), slides were incubated with primary antibodies for 30 minutes, followed by HRP-conjugated secondary antibodies for 10 minutes. Tyramide amplification (Perkin Elmer) was applied for 10 minutes. For multiplex staining, antigen retrieval and blocking were repeated. Single-stain controls were included. The primary antibodies used were Arg1 (Proteintech Group, Inc., 66129-1-Ig, 1: 400 dilution), Trem2 antibody (Abcam, ab305103, 1:500 dilution), F4/80 antibody (Proteintech Group, Inc., 29414-1-AP, 1:200 dilution), CD11c antibody (Servicebio, GB11059-100, 1:200 dilution). The imageries of the IF were captured using an Zeiss Axio Observer A1 microscope (Zeiss, Germany). Colocalization analysis was conducted using the ImageJ software on the Fiji platform.

Statistical analysis

Bioinformatic analyses were performed using R (v4.4.2) and Python (v3.12). Continuous variables are expressed as the mean ± standard deviation (SD) or mean ± standard error of the mean (SEM), as appropriate. Between-group comparisons were made using two-tailed Student's *t*-tests or, where appropriate, nonparametric Mann–Whitney U tests. For comparisons involving more than two groups, one-way or two-way analysis of variance (ANOVA) followed by Tukey's post hoc test was used. Ordinal variables, such as injury scores, were analyzed with the Kruskal–Wallis test, followed by Dunn's multiple comparisons test. Experimental data were statistically analyzed and visualized using GraphPad Prism 10 software. A p-value of < 0.05 was considered statistically significant.

Supplementary References

1. Hao, Y., et al., *Dictionary learning for integrative, multimodal and scalable single-cell analysis.* Nat Biotechnol, 2024. **42**(2): p. 293-304.doi:10.1038/s41587-023-01767-y
2. Korsunsky, I., et al., *Fast, sensitive and accurate integration of single-cell data with Harmony.* Nat Methods, 2019. **16**(12): p. 1289-1296.doi:10.1038/s41592-019-0619-0
3. Zappia, L. and A. Oshlack, *Clustering trees: a visualization for evaluating clusterings at multiple resolutions.* Gigascience, 2018. **7**(7).doi:10.1093/gigascience/giy083
4. Rudman-Melnick, V., et al., *Single-Cell Profiling of AKI in a Murine Model Reveals Novel Transcriptional Signatures, Profibrotic Phenotype, and Epithelial-to-Stromal Crosstalk.* J Am Soc Nephrol, 2020. **31**(12): p. 2793-2814.doi:10.1681/asn.2020010052
5. Melo Ferreira, R., et al., *Integration of spatial and single-cell transcriptomics localizes epithelial cell-immune cross-talk in kidney injury.* JCI Insight, 2021. **6**(12).doi:10.1172/jci.insight.147703
6. Balzer, M.S., et al., *Single-cell analysis highlights differences in druggable pathways underlying adaptive or fibrotic kidney regeneration.* Nat Commun, 2022. **13**(1): p. 4018.doi:10.1038/s41467-022-31772-9
7. Wang, W., et al., *Single-cell dissection of cellular and molecular features underlying mesenchymal stem cell therapy in ischemic acute kidney injury.* Mol Ther, 2023. **31**(10): p. 3067-3083.doi:10.1016/j.ymthe.2023.07.024
8. Love, M.I., W. Huber, and S. Anders, *Moderated estimation of fold change and dispersion for RNA-seq data with DESeq2.* Genome Biol, 2014. **15**(12): p.

550.doi:10.1186/s13059-014-0550-8

9. Trapnell, C., et al., *The dynamics and regulators of cell fate decisions are revealed by pseudotemporal ordering of single cells.* Nat Biotechnol, 2014. **32**(4): p. 381-386.doi:10.1038/nbt.2859

10. Jin, S., M.V. Plikus, and Q. Nie, *CellChat for systematic analysis of cell-cell communication from single-cell transcriptomics.* Nat Protoc, 2025. **20**(1): p. 180-219.doi:10.1038/s41596-024-01045-4

11. Fang, T., et al., *Anti-CD45RB Antibody Therapy Attenuates Renal Ischemia-Reperfusion Injury by Inducing Regulatory B Cells.* J Am Soc Nephrol, 2019. **30**(10): p. 1870-1885.doi:10.1681/asn.2018101067
